# Genome‐Wide Homozygosity Predicts Inbreeding Depression in the Hihi/Stitchbird (*Notiomystis cincta*) Better Than Realised Load

**DOI:** 10.1111/eva.70252

**Published:** 2026-05-21

**Authors:** Hui Zhen Tan, Katarina C. Stuart, Joseph Guhlin, Tram Vi, Selina Patel, Laura Duntsch, John G. Ewen, Patricia Brekke, Anna W. Santure

**Affiliations:** ^1^ School of Biological Sciences University of Auckland Auckland New Zealand; ^2^ Centre for Computational Evolution (CCE), Faculty of Science University of Auckland Auckland New Zealand; ^3^ Applied BioSciences Macquarie University Sydney New South Wales Australia; ^4^ Genomics Aotearoa, Biochemistry Department, School of Biomedical Sciences University of Otago Dunedin New Zealand; ^5^ Livestock Improvement Corporation Ltd Hamilton New Zealand; ^6^ Institute of Zoology Zoological Society of London London UK

**Keywords:** fitness, genetic load, hihi, homozygosity, inbreeding, inbreeding depression

## Abstract

Population declines result in increasingly small populations, which often experience an increase in inbreeding. Inbreeding may be negatively associated with fitness traits like survival and reproduction, that is, inbreeding depression, and is therefore detrimental to population persistence and adaptive potential. Realised genetic load—the count of homozygous deleterious alleles—is increasingly used as a proxy for fitness when investigating inbreeding depression. However, assessing the limitations of genetic load in predicting fitness, and comparisons with other homozygosity and inbreeding metrics, are needed. Our study system is the hihi/stitchbird ( *Notiomystis cincta* ), a forest bird endemic to Aotearoa, New Zealand. The hihi was extirpated from the North Island in the late 1800s and underwent a prolonged bottleneck on an island before reintroduction efforts began in 1980. In this study, we used imputed whole‐genome resequencing data of over 400 individuals spanning two decades from Tiritiri Matangi, the largest reintroduced population. We quantified inbreeding using runs of homozygosity (ROH), annotated variants using variant effect predictor, and detected variants related to lifetime reproductive success (LRS) through a genome‐wide association study (GWAS). We then tested for inbreeding depression by modelling LRS against genome‐wide homozygosity, homozygosity in coding regions, inbreeding (F_ROH_) and realised load. We found moderately high inbreeding levels in the hihi (mean F_ROH_ = 0.27). GWAS revealed two variants associated with LRS that are in or near genes associated with laying performance and yolk weight in other birds. We found genome‐wide homozygosity to best predict fitness, although F_ROH_ may be more useful at lower SNP densities. A relationship between realised genetic load and fitness likely reflects genome‐wide homozygosity levels, and high‐impact SNPs may present a misleading proxy for fitness. Our study provides support for genetic exchange to reduce inbreeding levels and contributes to a greater understanding of small population genetics and measures of inbreeding depression.

## 1 Introduction

Increased inbreeding is a major threat to the adaptive potential of small populations (Hohenlohe et al. 2021). This challenge will become increasingly relevant as more than half of all species are facing population decline (Finn et al. 2023). As populations become smaller, the number of potential mates in the population decreases, thereby increasing the likelihood of mating with a close relative. In certain mating systems, super‐breeders with exceptionally high breeding success may also have disproportionate genetic contributions to future generations, thereby increasing inbreeding (Nichols et al. 2024; Stein et al. 2017). Bottlenecks further contribute to inbreeding, with additional influences from stochastic processes like genetic drift, selection leading to purging of deleterious alleles and demographic history (Bouzat 2010).

A large body of research has demonstrated the negative effects of inbreeding on an individual's ability to contribute offspring to the next generation, that is, fitness (Wadgymar et al. 2024). This reduction in fitness caused by inbreeding is known as inbreeding depression (Charlesworth and Willis 2009). Inbreeding can be associated with reduced survival during embryo development (Hemmings et al. 2012; Nieminen et al. 2001; Phillippi and Yund 2017; Spottiswoode and Møller 2004), in juveniles (Duntsch et al. 2023; Hewett et al. 2024; Honan 2008; Zhao et al. 2016) and in adults (Coltman et al. 1999), and can have varying effects across these different life stages (Hewett et al. 2025b). Inbreeding is also associated with reduced reproductive success across a wide variety of species (Clark et al. 2025; Fang and Li 2023; Kyriazis et al. 2025b; Mitchell et al. 2025; Willoughby et al. 2019), including humans (Swinford et al. 2023). High levels of inbreeding may also lead to impaired cognition and aberrant behaviour, which impact survival and reproduction (Townsend et al. 2025). Overall, inbreeding is therefore detrimental to individuals and populations, although its effects vary depending on its interactions with other genomic and environmental factors (Hedrick and Kalinowski 2000). Mechanisms of inbreeding depression are increased homozygosity for recessive deleterious alleles (often described as ‘realised load’, discussed below) and increased homozygosity for alleles with heterozygote advantage (Charlesworth and Willis 2009; Kyriazis et al. 2021), although the relative contribution of both is poorly understood, especially in wild populations (Kardos et al. 2016). Inbreeding therefore threatens the long‐term success of populations (Keller and Waller 2002) and their adaptive potential due to the increased proportion of deleterious homozygous genotypes, along with its interplay with genetic drift, which reduces genetic diversity in the population (Femerling et al. 2023; Leroy et al. 2018; O'Grady et al. 2006).

Inbreeding and inbreeding depression have therefore become a significant concern for small populations, both wild and captive, including those in conservation programs (Keller and Waller 2002; Kristensen and Sørensen 2005; Leberg and Firmin 2008). Consequently, reducing inbreeding has become a priority for successful management globally (Hedrick and Kalinowski 2000). Genetic rescue refers to the deliberate introduction of beneficial variation from another population via immigrants (Hedrick and Garcia‐Dorado 2016; Tallmon et al. 2004), which has been shown to improve fitness via increased genetic variation and hybrid vigour (Frankham 2015; Whiteley et al. 2015). On the other hand, purging refers to the (usually) unintended reduction in deleterious variants that contributes to inbreeding depression (Wang 2000). Purging occurs through increased purifying selection on recessive deleterious variants, which are exposed due to increased homozygosity arising from inbreeding (Hedrick and Garcia‐Dorado 2016). Deliberate purging remains scarcely practised due to great uncertainty about its success and the potential harm to populations (Boakes et al. 2007; Leberg and Firmin 2008). In both genetic rescue and purging, a detailed understanding of inbreeding and inbreeding depression in the focal and source populations is nonetheless essential (Hedrick 1994; Hedrick and Garcia‐Dorado 2016).

With advances in sequencing technology and bioinformatic tools, inbreeding and inbreeding depression can now be quantified more precisely using genomics, even in the absence of a pedigree (Hammerly et al. 2016; Kardos et al. 2016). Inbreeding leads to higher homozygosity than expected by random mating, and in the absence of a pedigree, inbreeding depression has historically been tested using metrics such as multi‐locus heterozygosity at a small number of markers (Chapman et al. 2009; Grueber et al. 2008). However, whole‐genome marker resolution enables much finer‐scale dissection of inbreeding across the genome (Kardos et al. 2016). When close relatives mate, identical regions of the genome from a common ancestor are inherited by the inbred offspring, resulting in runs of homozygosity (ROH), which are continuous stretches of homozygous genotypes that are identical‐by‐descent due to inbreeding. Studies suggest that long ROHs, typically greater than 1 or 2 Mb in length and which reflect recent inbreeding, are a good indicator of fitness and inbreeding depression (Kyriazis et al. 2025a), and may be enriched for deleterious mutations (Szpiech et al. 2013). A growing number of studies have employed ROH metrics to quantify inbreeding and test for inbreeding depression in natural populations (Bérénos et al. 2016; Duntsch et al. 2021; Humble et al. 2020; Khan et al. 2021), including leveraging ROH to test for association to detect regions in the genome where being homozygous for one allele reduces fitness relative to the other genotypes (Duntsch et al. 2023; Hewett et al. 2025b; Stoffel et al. 2021a).

Inbreeding depression may also be investigated through the lens of genetic load, which refers to genetic variation that is deleterious and reduces the fitness of individuals (Bertorelle et al. 2022). Calculations of load can be trait‐based or molecular‐based (Plough 2016), but the latter has become increasingly popular, where deleterious mutations are predicted based largely on the evolutionary conservation of alleles or the functional effects of base substitution within coding regions (Bertorelle et al. 2022). Genome‐wide realised load can then be assessed as the total number of homozygous derived high‐impact variants (Bertorelle et al. 2022; Cavill et al. 2024; Dussex et al. 2021; Femerling et al. 2023; Lavanchy et al. 2024), or simply the sum of the number of rare homozygote high‐impact variants if derived and ancestral alleles cannot be identified (Stuart et al. 2024; Zhang et al. 2025).

Genomics has increased our capacity to study inbreeding depression, especially in non‐model organisms, thereby improving our understanding of the effects and nuances of inbreeding depression across species (Hoffman et al. 2014; Kyriazis et al. 2025a). However, we need to evaluate the effectiveness of different approaches—direct measures of fitness, genome‐wide inbreeding and genomic estimates of genetic load—in quantifying inbreeding depression, especially given the limited assessment of associations between genetic proxies of load and fitness to date (Schmidt et al. 2024). Such understanding is important for the development of the field, as direct measures of fitness for wild populations are prohibitive due to the need for intensive and long‐term monitoring (Bertorelle et al. 2022; Kardos et al. 2016; Parreira et al. 2025). Such comparisons will also enable researchers to understand the limitations of interpretation when using certain genetic proxies for fitness (Robledo‐Ruiz et al. 2025).

Our study system is the hihi/stitchbird ( *Notiomystis cincta* ), a forest bird endemic to the North Island of Aotearoa New Zealand. Once widespread across the North Island, hihi underwent a drastic population bottleneck in the 1800s due to predation by introduced mammals, habitat loss, and possibly disease (Taylor et al. 2005). The hihi became extinct from the North Island, surviving as a single population on Te Hauturu‐o‐Toi (36.1946° S, 175.0753° E), a pest‐free island (Brekke et al. 2011; Taylor et al. 2005). After almost a century of isolation on Te Hauturu‐o‐Toi, the translocation of hihi to establish reintroduced populations began in 1980 and remains an important conservation strategy for hihi today (Miskelly and Powlesland 2013). The drastic and prolonged population bottleneck, as well as the fact that reintroduced populations are small with little genetic exchange between them, has resulted in low genetic diversity, high inbreeding and little adaptive potential in the hihi (de Villemereuil et al. 2019; Duntsch et al. 2023; Lee et al. 2022).

In this study, we characterise inbreeding, inbreeding depression and genetic load in the hihi, and leverage whole genome resequencing data and life history information to (1) investigate inbreeding depression using both inbreeding and realised genetic load, (2) test for association between fitness and genome‐wide SNPs and (3) validate the use of genomic load as a proxy for fitness in wild populations.

## 2 Materials and Methods

### 2.1 Sample Collection and Sequencing

Our study focuses on hihi individuals sampled as part of long‐term conservation efforts on the island of Tiritiri Matangi (36.6012° S, 174.8894° E). We selected individuals for whole genome resequencing with the aim of including samples from all Tiritiri Matangi cohorts spanning 2001/02–2022/23 (*n* = 420). A cohort refers to individuals born within the same breeding season (Austral spring and summer). We further included birds from two additional populations, Te Hauturu‐o‐Toi (*n* = 27; remnant population) and Zealandia Te Māra a Tāne Wildlife Sanctuary (41.2944° S 174.7500° E; *n* = 30), due to genealogical relationships with Tiritiri Matangi individuals and as part of a broader study (Figure S1). Of the Te Hauturu‐o‐Toi birds, 20 were translocated to Tiritiri Matangi in 2010 as population top‐ups, five were sampled in 2010 as well but were not translocated, and two were the reference genome individuals sampled in 2017 and 2018, respectively (Bailey et al. 2025). Blood samples were collected from all Tiritiri Matangi and Te Hauturu‐o‐Toi birds via brachial venipuncture and stored in 95% ethanol at −30°C. For Zealandia individuals, 2–3 downy feathers were plucked from the underside of 21‐day‐old nestlings and stored in 95% ethanol at −30°C. DNA was extracted from both blood and feather samples using the DNeasy Blood & Tissue Kits (Qiagen) following the manufacturer's protocols. Whole‐genome sequence (WGS) libraries were prepared by AgResearch, New Zealand in two batches. We sequenced a total of 477 samples across the two batches. The first batch consisted of 322 samples where libraries with high DNA input were prepared using the Illumina DNA Prep kit (Illumina), while libraries with low DNA input were prepared using the Nextera XT kit (Illumina). This batch included 31 samples, previously published in Stuart et al. (2024), that were sequenced at high coverage of ~20X for use as reference panel individuals for imputation (*n* = 30 following sample quality control; Tan et al. 2025; Vi et al. 2025). We selected these reference panel individuals based on their high lifetime total number of offspring and, therefore, representation of population haplotype diversity. The remaining samples were sequenced at low coverage (~3X; Vi et al. 2025). The second batch consisted of 158 samples, including 3 duplicated samples from the first batch. Libraries were prepared using the NEB Next Ultra II FS DNA Library Prep Kit for Illumina (New England Biolabs), where the PCR‐free version was used for high‐input samples while PCR was used for low‐input samples. DNA libraries in both batches were sequenced on the Illumina NovaSeq 6000 platform, which produces 150 bp paired‐end reads.

### 2.2 Processing Raw Sequences

Following our previous approach (Stuart et al. 2024; Tan et al. 2025; Vi et al. 2025), raw sequences for all 477 sequenced individuals were first processed using TrimGalore v0.6.7 (Krueger et al. 2021) with default parameters to remove Illumina adaptors and to trim bases with Phred score below 20. FastQC files were generated within TrimGalore (–fastqc) and aggregated using MultiQC v1.13 (Ewels et al. 2016) for verification of sequence quality. As some samples with low DNA input had multiple DNA library replicates, we compiled reads across all library preparation runs per sample and merged them into a single fasta file for each read direction. We then aligned reads to the high‐quality female hihi reference genome (Bailey et al. 2025; Aotearoa Genomic Data Repository, Project Code NZ‐00034, Dataset ID AGDR00034, https://doi.org/10.57748/ZD00‐D451) using default settings in BWA‐MEM (Li and Durbin 2009). We sorted our alignments (sort), converted them into BAM files (view) and generated alignment statistics (flagstat) using SAMtools v1.15.1 (Li et al. 2009). Duplicates were identified and marked using MarkDuplicates in picard v2.26.10 (Broad Institute 2019). The final alignment file was then indexed using SAMtools (index).

### 2.3 SNP Calling

We used HaplotypeCaller in GATK v4.6.1 (Poplin et al. 2017; Van der Auwera and O'Connor 2020) to generate a Genomic VCF (GVCF) per sample using the GVCF reference confidence model (‐ERC GVCF). We then merged all individual GVCF files to produce a population VCF (pVCF) file using GLnexus (Yun et al. 2020). We first used VCFtools v0.1.15 to remove SNPs that were multiallelic or with a mean depth > 50, and set genotypes with depth < 2 as missing. We then set half‐calls in the pVCF file to missing using BCFtools v1.19 (+setGT; Li 2011) and filtered the pVCF file to remove sites marked as ‘monoallelic’ by GLnexus and any SNPs with QUAL < 40 also using BCFtools v1.19. A total of 5,984,615 SNPs were retained, and the average site missingness across all individuals was 0.253. Following SNP calling, we calculated allele frequency and per‐sample heterozygosity using VCFtools v0.1.15 (Danecek et al. 2011; Figure S2). One of the high‐coverage Tiritiri Matangi individuals was removed due to high heterozygosity, as observed in previous studies that used the same set of high‐coverage samples (Stuart et al. 2024; Tan et al. 2025; Figure S2).

### 2.4 Sample Checks

Sample mix‐ups have happened within the hihi dataset before, which is likely due to samples being duplicated, mislabelled or swapped. As such, we followed the framework of Duntsch et al. (2022) to perform sample checks to ascertain the identity of samples, that is, confirm samples are from the individual that we think they are from. We achieved this by assessing the concordance between sample information, specifically relatedness and sex, as obtained from the pedigree and our genetic data. This sample check process was only performed for 410 individuals born on Tiritiri Matangi, excluding offspring of a mating event between a Te Hauturu‐o‐Toi and Tiritiri Matangi bird (‘F1’ individuals). All individuals in the population are banded at ~21 days (1 week before fledging) and their parents are genetically verified using a panel of microsatellite markers, enabling full pedigree reconstruction (Brekke et al. 2012; de Villemereuil et al. 2019). For relatedness, we calculated pedigree relatedness using kinship2 (Sinnwell et al. 2014) in R 4.3.2 (R Core Team 2023) and whole‐genome genetic relatedness using PLINK 2.0 (–make‐rel) (Chang et al. 2015) with a MAF filter of 0.1 applied. We then plotted pedigree relatedness against genetic relatedness to compare their concordance (Figure S3).

We noted that two samples were consistently involved in discordant pairwise relationships between pedigree and genetic relatedness (Figure S4). Upon inspection, both individuals showed very low genetic relatedness to their known parents (maximum relatedness of 0.076 while > 0.3 was expected). Both samples had very high missingness (0.96 and 0.93, respectively), suggesting that the sequences were of very poor quality, which would impact downstream analyses. We therefore removed these samples from our dataset.

For the remaining 408 samples, we first inspected known parent–child relationships (Figure S3a) and identified seven discordant pairwise relationships involving 11 unique individuals. For each of these 11 individuals, we checked their genetic relatedness to their parents and offspring that had also been sequenced; on average, 2.18 first‐degree kin were available per individual for checks. First‐degree kin relationships with genetic relatedness > 0.3 were marked as ‘verified’ while those < 0.1 were marked as ‘unverified’ (there were no relationships with genetic relatedness of 0.1–0.3). Only samples with at least one ‘verified’ relationship were retained for downstream analyses. Of the 11 individuals involved in outlier pairwise relationships, five were unverified and removed from downstream analyses. The same verification approach was also applied to discordant half‐sib/grandparent–grandchild/uncle/aunt to nephew/niece relationships (pedigree relatedness = 0.25; Figure S3b) and to unrelated pairs (pedigree relatedness of ~0; Figure S3c), which led to the removal of two more unverified samples. As an individual could be implicated in many outlier relationships across multiple relationship classes, performing verification sequentially across classes reduced the number of outlier points to check in subsequent classes if problematic individuals had already been removed. We further updated three parent–child and one full‐sib relationships in the pedigree, which improves concordance between genetic relatedness and pedigree‐relatedness such that the samples are now verified.

For sample checks on sex, we calculated the heterozygosity of all SNPs on the Z chromosome using VCFtools v0.1.15 (Danecek et al. 2011). In birds, females are the heterogametic (ZW) sex, while males are homogametic (ZZ), hence male birds should have higher heterozygosity in SNPs on the Z chromosome and vice versa as an indication of genetic sex. We arranged the samples in order of heterozygosity in Z and saw that, in general, most of our samples followed the expected trend where heterozygosity of samples recorded as females is usually < 0.04 while that for males can range from ~0.06 to 0.23 (Figure S5). Samples were identified as showing disagreement between genetic sex and recorded sex when their heterozygosity levels were significantly outside the expected range, for example, an individual recorded as female had a heterozygosity of 0.12, which is higher than the male average. Eight samples were identified as showing disagreement, including four unverified samples from the above relatedness checks. For the remaining four samples, we were able to verify their identity using the above relatedness checks. Using information from subsequent resighting information, we were able to revise the recorded sex for one of these samples. For the remaining three samples, their recorded sex might have been erroneous due to misidentifications of sex based on juvenile plumage or misidentifications of leg band colour combinations in the field leading to the observed sex being attributed to another individual. As they were verified in the relatedness checks, we were confident in retaining them for downstream analyses.

We updated the plot of pedigree relatedness against genetic relatedness (Figure S3) to include only verified individuals and confirmed that there was greater concordance. The remaining variation in genetic versus pedigree relatedness is likely due to differences between the proportion of the genome expected to be identical‐by‐descent based on pedigree and variation introduced during actual gamete formation that is controlled by genomic architecture such as recombination (Veller et al. 2020), and to some extent also familial relationships that were not documented in our long‐term pedigree, and missingness.

A total of nine samples were removed following sample checks, leaving 401 verified individuals for downstream analyses. We conducted principal component analysis (PCA) using PLINK 2.0 (Chang et al. 2015) to confirm that there were no indications of batch effects due to multiple batches of library preparation (Figure S6).

### 2.5 Genotype Imputation

The sample‐filtered population VCF file (401 samples in total) was then further variant‐filtered to prepare for imputation. We only retained SNPs on autosomal chromosomes.

#### 2.5.1 Reference Panel

A reference panel refers to the set of individuals, often sequenced at high coverage, selected with the aim of representing the diversity of haplotypes within a population (Das et al. 2018). We first subset the 30 high‐coverage samples using BCFtools v1.19 and quantified site and sample missingness using PLINK 2.0 (Chang et al. 2015). We then used BCFtools v1.19 to set genotypes with depth < 4 to missing (+setGT), and discard SNPs with > 5% missingness (B. Browning, pers. comms., 6 June 2024) as well as non‐variant sites. The final number of SNPs in our reference panel is 2,768,421. Samples were renamed using BCFtools v1.19 (Li 2011) with a ‘_R’ suffix to avoid issues of duplicated names between the reference and target panel, since high‐coverage samples were also included in the target panel (see next section).

#### 2.5.2 Target Panel

The target panel refers to the set of individuals whose genotypes are to be imputed (Browning et al. 2018). For the target panel, we included all 401 verified Tiritiri Matangi samples and quantified site and sample missingness using PLINK 2.0 (Chang et al. 2015). We included our 26 reference panel Tiritiri Matangi birds in this target set to impute the small number of their missing genotypes (< 5%). We then used BCFtools v1.19 (Li 2011) to set genotypes with depth < 4 to missing (+setGT), and discard SNPs with >40% missingness as well as non‐variant sites. The final number of SNPs in our target set is 1,628,353.

#### 2.5.3 Imputation Using Beagle v5.4

Our previous work demonstrated that Beagle v5.4 (Browning et al. 2018, 2021) provides high accuracy imputation, as assessed against a ground truth dataset using both a leave‐one‐out approach on our resequencing data and when comparing to SNPs genotyped on a SNP chip (Tan et al. 2025; Vi et al. 2025). We first phased our reference panel using Beagle v5.4 following default settings except with sliding windows of 60 cM (window = 60) and 24 iterations (iterations = 24). We then imputed using Beagle v5.4 following default settings except with sliding windows of 60 cM (window = 60), 1600 model states (imp‐states = 1600), and a minimum haplotype length of 6 cM (imp‐segment = 6) for haplotype segments to be included in the hidden Markov model (HMM). This choice of phasing and imputation parameters was informed by the combination that resulted in the highest imputation accuracy based on our previous leave‐one‐out tests (Tan et al. 2025).

#### 2.5.4 Post‐Imputation Filtering

We performed filtering on the imputed dataset to remove SNPs that were not reliably imputed. In the absence of ground truth genotypes, Beagle's estimated dosage R‐squared (DR2) was used as our metric for imputation accuracy. Beagle DR2 represents the estimated squared correlation between the imputed and true unobserved allele dose, calculated from the posterior genotype probabilities, as a measurement of uncertainty for imputation quality (Browning and Browning 2009; Pook et al. 2020; Ramnarine et al. 2015). SNPs with DR2 < 0.8 were removed (Figure S7), resulting in a high‐accuracy imputed dataset of 2,337,101 SNPs (84% of the reference SNPs).

### 2.6 Runs of Homozygosity

To quantify runs of homozygosity (ROH), we used RZooRoH 0.4.1 which applies HMM and a model‐based approach allowing multiple homozygous‐by‐descent (HBD) classes (Bertrand et al. 2019). RZooRoH shows more consistent results across varying coverage levels and performed better than rule‐based approaches with low SNP density datasets (Bertrand et al. 2019; Duntsch et al. 2021). Overall, the HMM approach is particularly beneficial in situations encountered in wild organisms including lower marker densities, low‐fold sequencing data, uneven marker spacing and variable recombination rates (Bertrand et al. 2019). We used a 13‐class model (K = 13) that has been shown to work best for the hihi dataset and is capable of capturing the smallest ROH segment (Duntsch et al. 2021). The 13 HBD classes are 10, 20, 30, 40, 50, 100, 200, 500, 600, 700, 1000, 2000, 2000, which represent different categories of HBD segment lengths, where the final class (the second ‘2000’) represents the non‐HBD class. We used the imputed genotype probability as input to improve accuracy by accounting for uncertainty in imputed genotypes (Tan et al. 2025). We first calculated allele frequencies across the filtered post‐imputation dataset using VCFtools v0.1.15 (Danecek et al. 2011), which allowed us to parallelise our RZooRoH runs, with each sample analysed individually. We then converted genotype probabilities for each sample from the VCF format into the gen format using BCFtools v1.19 (Li 2011). We used zoodata in RZooRoH to import our gen format file and allele frequency file and ran that using zoorun with our 13‐class model. From the output, we discarded HBD segments < 300 kb in length to remove short ROH arising from population linkage disequilibrium (Duntsch et al. 2021; Meyermans et al. 2020); this threshold removes SNPs with r^2^ > ~0.15 (K. D.Lee et al. 2022). We calculated the genomic inbreeding coefficient (F_ROH>300kb_, F_ROH_ hereafter) for each sample by summing the lengths of HBD segments per individual and dividing that by the total length of the hihi autosomal genome (~915 Mb; Bailey et al. 2025). We visualised the distribution of F_ROH_ values and ROH lengths using ggplot2 (Wickham 2016). For 268 individuals shared between this study and Duntsch et al. (2023), we visualised respective F_ROH_ and mean ROH values of each sample derived from both studies and calculated the correlation of values between studies using Kendall's τ (tau) with the cor.test function in R. We also visualised ROH density (proportion of individuals in ROH; Stoffel et al. 2021a) for non‐overlapping 500 kb windows using windowscanr v0.1 (Tavares 2025) and ggplot2, and calculated the correlation between ROH density and recombination rate for each window (obtained from Tan et al. 2024) using Kendall's τ (tau) as above.

### 2.7 Fitness Measure

We measure fitness in this study using lifetime reproductive success, defined as the total number of offspring an individual has over its lifetime—a commonly used metric that reflects both the production of offspring and their survival across reproductive seasons (Alif et al. 2022; Koch and Narum 2021). We were able to obtain lifetime reproductive success information for the Tiritiri Matangi population owing to dedicated and long‐term monitoring by conservation officers and volunteers, as well as long‐term genotyping efforts for pedigree construction. The total number of offspring per individual was calculated from the genetically verified Tiritiri Matangi pedigree (Brekke et al. 2012; de Villemereuil et al. 2019), and counts all offspring surviving to banding at ~21 days, a close proxy for fledging at ~28 days. For all analyses using lifetime reproductive success as a trait, we used a subset of our Tiritiri Matangi dataset. To minimise the impact of population substructure and heterosis (where offspring of genetically distinct individuals have greater fitness) on our analyses of inbreeding, load and inbreeding depression, we first excluded all F1 offspring of Te Hauturu‐o‐Toi and Tiritiri Matangi individuals (*n* = 9), leaving a dataset of 401 individuals. We then subset this dataset further to only include individuals that had completed their reproductive lifespan, as assessed by not having been detected in the subsequent two population surveys post‐fledging of the most recent cohort (2022/23) in our dataset (i.e., surveys in September 2023 and February 2024) or for whom there was no evidence of breeding for the past two breeding seasons. The final number of birds in this dataset is 347.

### 2.8 Quantifying Genetic Load

We explore two methods for detecting variants that reduce fitness, that is, contribute to genetic load. First, we conducted a genome‐wide association study (GWAS) with lifetime reproductive success as our trait of interest. Then, we also ran Ensembl Variant Effect Predictor (McLaren et al. 2016) to identify high‐impact variants.

#### 2.8.1 Genome‐Wide Association Study

We used individuals with complete lifetime reproductive success data (*n* = 347) for our GWAS. We applied mixed linear model (MLM) association analyses in TASSEL 5.2.94 (Bradbury et al. 2007), which runs both additive and dominant models. MLM is useful in accounting for population structure and kinship within GWAS (Z. Zhang et al. 2010). We applied a MAF filter of 0.05 to our high‐accuracy imputed SNP genotypes, resulting in 2,107,359 input SNPs for TASSEL. We ran TASSEL on each chromosome separately. We used lifetime reproductive success as the phenotype, F_ROH_ as the covariate, and cohort and sex as factors in our input phenotype file. Within TASSEL, we used –intersect to join phenotype and genotype input datasets based on sample name. We also accounted for kinship by supplying a genome‐wide kinship matrix calculated in TASSEL using the centred identity by state (IBS) method. From the output of TASSEL, we removed any locus with at least one genotype having < 5 observations, since its effect estimate may be unreliable due to outliers (Bradbury et al. 2021). GWAS results from TASSEL were visualised with topr 2.0.2 (Juliusdottir 2023). Regions of the genome associated with lifetime reproductive success were identified by the presence of highly significant SNPs, which are SNPs at or near the threshold of −log_10_(*p*) > 7.62, representing *p* < 0.05 with Bonferroni correction (0.05/2,107,359). As our dataset does not fulfil TASSEL's assumption of a normally distributed error distribution, we further evaluated the two most significant SNPs by fitting MCMCglmm models (Hadfield 2010) in R 4.3.2 for each SNP separately. Lifetime reproductive success was modelled with SNP genotypes and sex as fixed factors. Cohort, maternal ID and genetic relatedness were included as random effects. We evaluated the support for individual fixed effects using pMCMC (Hadfield 2010). All models were run using a zero‐inflated Poisson error distribution due to an excess of zero counts in our lifetime reproductive success data (Figure 3), and following de Villemereuil et al. (2019). Runs were checked for convergence using the heidel.diag function in coda 0.19‐4 (Plummer et al. 2006).

To detect genes associated with differences in lifetime reproductive success, we looked for genes within 50 kb (r^2^ > ~0.2; Lee et al. 2022) of the two most significant SNPs and searched the literature for studies showing associations between those genes and traits related to reproduction, especially in birds. We also created a dataset of 298 genes containing strongly associated SNPs (raw *p*‐values < 0.0001; *n* = 1,460) to test whether any gene classes are overrepresented in regions showing higher association with lifetime reproductive success relative to a reference list of genes on the chromosome‐level assembly of the hihi. Gene overrepresentation tests were performed using the web tool of PANTHER v19.0 (Mi et al. 2019; Thomas et al. 2022) using the chicken ( *Gallus gallus* ) reference proteome, applying Fisher's exact test with false discovery rate (FDR) correction. We performed the test for biological, molecular and cellular annotation datasets of both GO (gene ontology) and PANTHER GO‐Slim. GO terms are significantly overrepresented if their *p*‐value and FDR are < 0.05.

#### 2.8.2 Variant Effect Predictor

We used all verified Tiritiri Matangi individuals in this analysis (*n* = 401). We predicted the effects of the coding region variants in the hihi using Ensembl Variant Effect Predictor (VEP) v113.3 (McLaren et al. 2016). We started by polarising our SNPs from the high‐accuracy imputed dataset with no MAF filters applied. We utilised our previous Progressive Cactus (Armstrong et al. 2020) alignment between the hihi reference genome and other passerine genomes of Eastern Yellow Robin ( *Eopsaltria australis* ; GCA_034509425.1), Barn Swallow ( *Hirundo rustica* ; GCF_015227805.2), Zebra Finch ( *Taeniopygia guttata* ; GCF_003957565.2) and House Sparrow ( *Passer domesticus* ; GCF_036417665.1), with Chicken ( *Gallus gallus* ; GCF_016699485.2) as the outgroup (Stuart et al. 2024). The reconstructed genome of the ancestor of the ingroup species, Anc01, was inferred as the ancestral state for hihi.

Using halAncestralAlleles (Guhlin et al. 2026; https://github.com/jguhlin/hal), we extracted the ancestral allele from the Anc01 ancestral sequence in the Progressive Cactus alignment for each SNP position in our dataset of high‐accuracy imputed genotypes. The inferred ancestral allele (AA) for each SNP fell into four categories relative to our pVCF's reference/alternate alleles (REF/ALT), which we describe here alongside the percentage of SNPs within each category given in brackets, followed by how we formatted our pVCF's REF/ALT alleles into the VEP format. Category 1: AA match REF (54.7%)—retain REF/ALT for input. Category 2: AA match ALT (35.7%)—swap the REF/ALT bases for input. Category 3: no AA reconstructed (5.7%)—retain REF/ALT for input. Category 4: novel AA (does not match REF or ALT; 3.9%)—split each position into two entries with both the original REF/ALT alleles as the ALT alleles of each respective entry, that is, AA/REF and AA/ALT (see Table S1 for a summary). In addition to formatting the VEP input file, we also replaced the corresponding REF alleles of Categories 2 and 4 in the hihi reference genome using maskfasta in BEDTools v2.31.1 (Quinlan and Hall 2010) to reflect the new ancestral allele. We also removed duplicated isoforms in our genome annotation file by retaining the longest isoforms using agat_sp_keep_longest_isoform.pl in AGAT v1.0.0 (Dainat 2022). VEP was run with the ancestral and derived alleles formatted in the default Ensembl VEP input format, modified hihi reference genome assembly and annotation file retaining the longest isoforms as input. Results were output for variants in coding regions only. We visualised the distribution of derived MAF for SNPs per impact class using ggplot2. We also performed gene overrepresentation tests on the list of genes containing high‐impact variants (97 genes containing 258 SNPs), using the same method employed for GWAS SNPs.

### 2.9 Modelling Inbreeding Depression

Using the same set of 347 individuals with complete lifetime reproductive success information that were utilised in our GWAS above, we modelled the relationship between genomic inbreeding (F_ROH_) and fitness (i.e., lifetime reproductive success) to characterise inbreeding depression in the hihi using MCMCglmm (Hadfield 2010) in R 4.2.1. Lifetime reproductive success was modelled with F_ROH_ and sex, as well as their interaction, as fixed effects. Cohort and maternal ID were included as random effects. These models were also run using the zero‐inflated Poisson error distribution due to an excess of zero counts in our lifetime reproductive success data (Figure 3), and following de Villemereuil et al. (2019). Runs were checked for convergence using the heidel.diag function in coda 0.19–4 (Plummer et al. 2006).

While homozygote counts for high‐impact mutations have been used as a genetic load proxy for fitness (Grossen et al. 2020—Alpine ibex [ *Capra ibex* ]), Robledo‐Ruiz et al. (2025) found that inbreeding metrics such as F_ROH_ predict fitness better than genetic load proxies in a dataset of Helmeted honeyeaters ( *Lichenostomus melanops cassidix* ). Inspired by Robledo‐Ruiz et al. (2025), we performed similar investigations for hihi across various metrics measuring genome‐wide homozygosity, genetic load and inbreeding (F_ROH_), allowing us to assess their relative effectiveness in detecting inbreeding depression. For genome‐wide homozygosity, we calculated (1a) the proportion of homozygous sites and (1b) the number of sites homozygous for derived alleles across the genome. For genetic load, we counted, per individual, the number of sites homozygous for derived (2a) high‐impact alleles, (2b) moderate‐impact alleles, and (2c) low‐impact alleles. For inbreeding, we calculated (3a) raw F_ROH_ values (without removing ROH< 300kb), (3b) F_ROH>300kb_ (see Materials and Methods: Runs of homozygosity) and (3c) F_ROH>1Mb_. We tested the association of each of these eight metrics against fitness (lifetime reproductive success) using the MCMCglmm model described above, but without the interaction term. All MCMCglmm models were assessed using their deviance information criteria (DIC), and support for individual fixed effects was evaluated using pMCMC (Hadfield 2010). Pairwise correlations between all eight metrics were visualised and tested using ggpairs in GGally (Schloerke et al. 2025) in R 4.3.2.

## 3 Results

### 3.1 Moderately High Genomic Inbreeding in the Tiritiri Matangi Population

**FIGURE 1:**
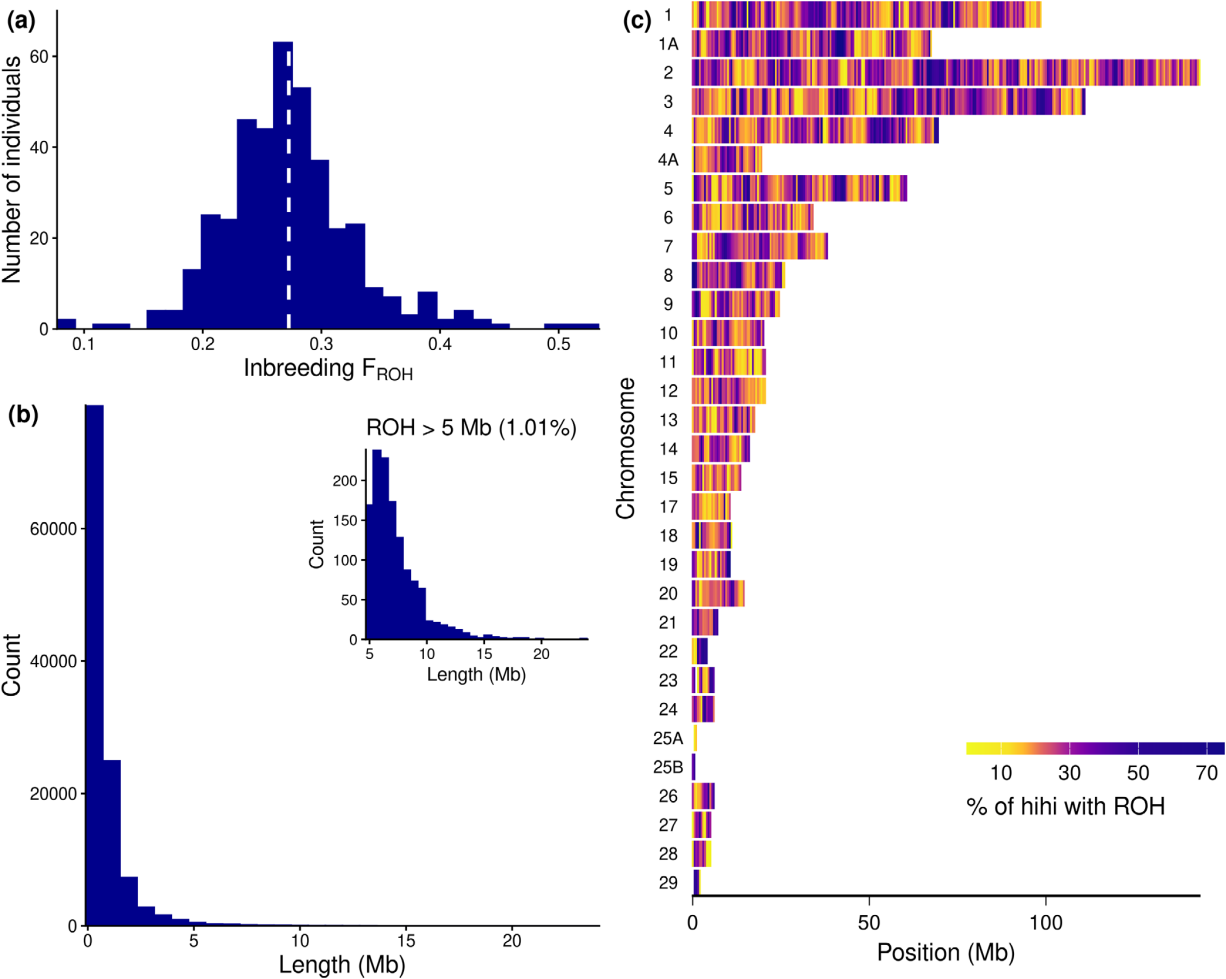
Runs of homozygosity (ROH) in 401 hihi/stitchbird from Tiritiri Matangi. (a) Histogram showing distribution of F_ROH_ (proportion of genome in ROH > 300 kb) with the white dotted line showing mean F_ROH_ of 0.27 (median = 0.27, SD = 0.06). (b) Histogram showing distribution of ROH lengths for all ROH > 300 kb (mean = 0.85 Mb, median = 0.51 Mb, SD = 1 Mb). The inset shows the distribution of ROH lengths for very long ROH (> 5 Mb). (c) Genome‐wide ROH density in non‐overlapping 500 kb windows along each autosomal chromosome in the hihi genome. ROH density represents the proportion of individuals in ROH within each window.

We found moderately high levels of inbreeding in the Tiritiri Matangi population with considerable variation in genome‐wide inbreeding measured by F_ROH>300kb_ (F_ROH_ hereafter) values within the population (mean and median = 0.27, SD = 0.06; Figure 1a). The three most inbred individuals, with F_ROH_ values of ~0.5, include two individuals with F_ROH_ > 0.5 that were full‐siblings from a mother‐son mating event and one individual with F_ROH_ = 0.494 from a father‐daughter mating event. The fourth most inbred individual had F_ROH_ = 0.457 from a full‐sibling mating event. All other individuals had F_ROH_ of < ~0.42.

A large majority of ROH (~92.5%) were very short (< 300 kb) and were excluded from downstream analyses. For the retained ROH > 300 kb, 79% of ROH are < 1 Mb, and only ~1% of ROH are > 5 Mb in length (mean = 0.85 Mb, median = 0.51 Mb, SD = 1 Mb; Figure 1b). Compared to Duntsch et al. (2023), we recovered shorter ROH lengths on average despite very similar F_ROH_ results (Figure S8). We observed variation in ROH density across the genome and higher ROH density in macrochromosomes (Figure 1c). High ROH density at some chromosome ends, such as in chromosomes 4 and 8, was unexpected given that avian chromosome ends tend to have high recombination rates (Backström et al. 2010; Peñalba et al. 2020; Tan et al. 2024). However, more generally, high ROH density corresponds to regions of low or suppressed recombination, for example, the midpoint of chromosome 8 (Figures 1c and S9; Tan et al. 2024). When ROH density and sex‐averaged recombination rates were compared across non‐overlapping 500 kb intervals, we found a significant but weak negative correlation (Kendall's tau = −0.203, *p*‐value < 2.2e‐16; Figure S9).

### 3.2 Genetic Load

#### 3.2.1 Genome‐Wide Association Study

**FIGURE 2:**
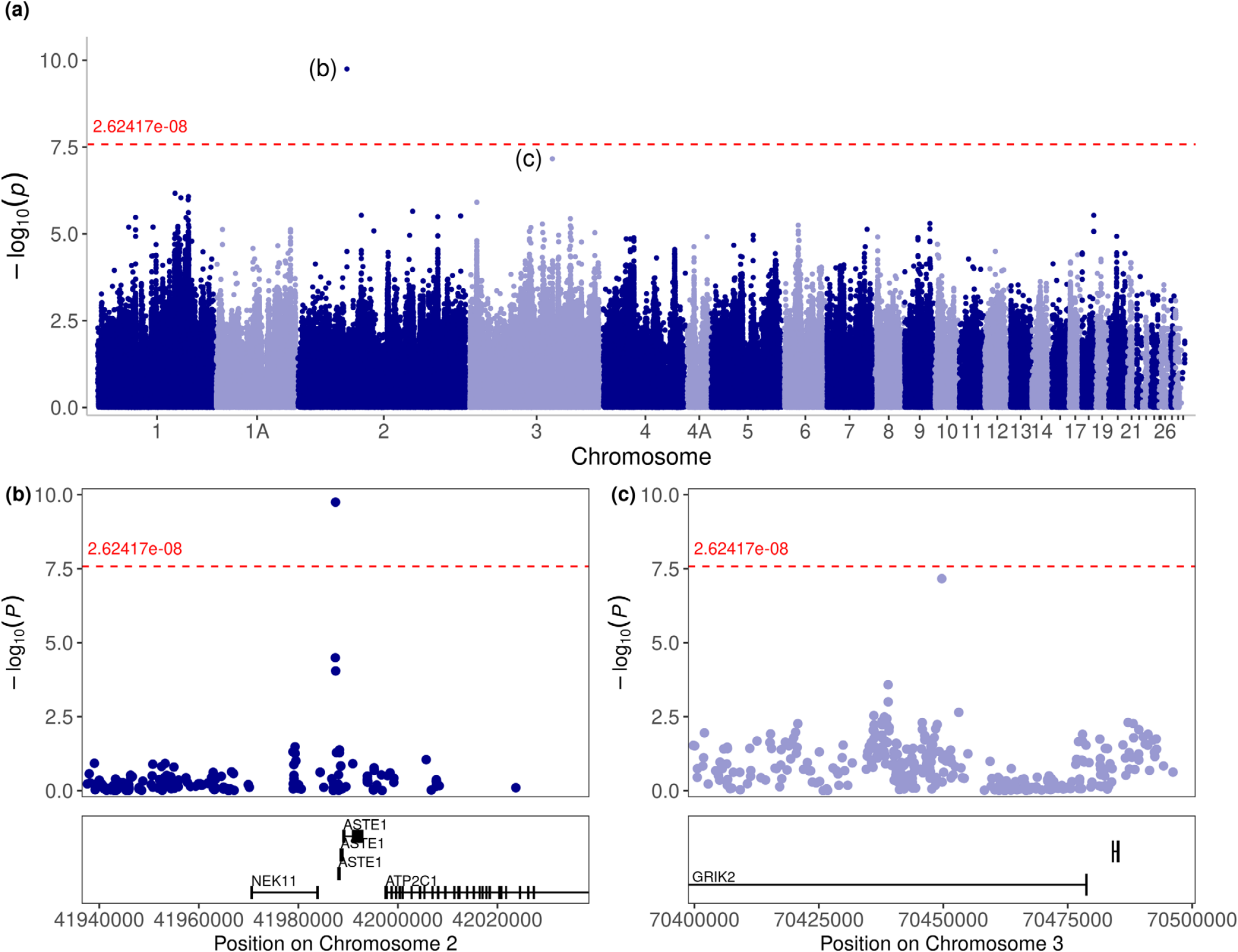
Genome‐wide association study (GWAS) of SNP effects on the lifetime reproductive success of 347 hihi/stitchbird individuals from Tiritiri Matangi. Lifetime reproductive success is the total number of offspring an individual has over its lifetime and was only calculated for individuals who have completed their reproductive lifespan. (a) Manhattan plot of GWAS results from the dominant model show a significant SNP on chromosome 2 and a SNP on chromosome 3 that is slightly below the significance threshold after Bonferroni correction (−log_10_(p) > 7.62), as represented by the red dashed line. The SNPs of interest next to the labels (b) and (c) are those featured in the zoomed‐in regions in the bottom half of the figure. Axis ticks after chromosome 21 represent chromosomes 22, 23, 24, 25A, 25B, 26 (labelled in figure), 27, 28, 29. (b) Zoomed‐in region of +/− 50 kb around the significant SNP on chromosome 2 at 41,987,466 bp that is close to genes NEK11, ASTE1 and ATP2C1. (c) Zoomed‐in region of +/− 50 kb around a marginally significant SNP on chromosome 3 at 70,449,698 bp, which overlaps gene GRIK2. Black rectangles in (b) and (c) present the exons.

Inbreeding depression is hypothesised to be a result of inbreeding increasing the frequency of homozygosity at recessive deleterious alleles, and/or increasing homozygosity for alleles with heterozygote advantage, both of which represent cases of non‐additive allele effects. We therefore focus on the dominant model in our GWAS to explicitly test for differences in fitness between the three genotypes. Our GWAS results from the dominant model suggest that inbreeding depression is likely to be polygenic in the hihi, with contributions from multiple regions across the genome. Two genomic loci that could be involved are on chromosomes 2 and 3. In chromosome 2, one SNP at 41,987,466 bp is significantly associated with lifetime reproductive success after Bonferroni correction (Figure 2a,b; Table S2). At this SNP, we observed 306 individuals homozygous for the major allele, 10 homozygous for the minor allele and 31 heterozygous (Table S2). The significant SNP did not overlap any genes but is in proximity to ASTE1, NEK11 and ATP2C1 (at distances of 524, 3527 and 9996 bp, respectively), although genotyped SNPs overlapping those genes showed no signals of association with lifetime reproductive success. In chromosome 3, we additionally identified another SNP, at 70,449,698 bp, that is only slightly below the significance threshold, indicating potential for association (Figure 2a,c; Table S2), especially considering our stringent significance threshold (Fadista et al. 2016). At this SNP, we observed 302 individuals homozygous for the major allele, 22 homozygous for the minor allele and 23 heterozygous (Table S2). This SNP on chromosome 3 overlapped with GRIK2. The GWAS results were supported by separate MCMCglmm models where the genotype at both SNPs was a significant predictor of the count component of lifetime reproductive success, although we acknowledge the low sample sizes used (Tables S3–4). Genes in these two focal regions are associated with reproductive traits in other birds such as laying rate (ATP2C1, GRIK2; Bhavana et al. 2022; Reyer et al. 2021), yolk weight (GRIK2; Gao et al. 2023) and a variety of important biological functions, such as development (ANO10; Pritchett et al. 2017), immunity (NEK11; Strillacci et al. 2019) and plumage (ATP2C1; Nie et al. 2016). In the additive model, one SNP at 64,981,067 bp on chromosome 2 was significantly associated with lifetime reproductive success, alongside peaks in chromosomes 1, 5, 7 and 9 (Figure S10).

For the dominant model, significant gene overrepresentation amongst the 298 genes containing strongly associated SNPs was detected for three GO terms in the GO‐Slim Biological and GO‐Slim Molecular annotation dataset (Table S5). These three GO terms are negative regulation of chondrocyte proliferation (GO:1902731), transforming growth factor beta receptor activity (GO:0005024) and transmembrane receptor protein kinase activity (GO:0019199).

#### 3.2.2 Variant Effect Predictor

Our investigation into coding region SNPs across the hihi genome that contribute to genetic load revealed that more than half of these are synonymous variants that have low functional impacts (Table 1). The next most common variant type is missense variants that are predicted to have moderate functional impacts. Two hundred and fifty‐eight SNPs (0.87%) are predicted to have high functional impact by changing the start or stop codons. Higher impact SNPs tend to have lower minor allele frequency (MAF) on average, although the MAF distributions largely overlap (Figure S11). When plotted against the dominant‐model GWAS, high‐impact SNPs do not appear to co‐locate with variants associated with lifetime reproductive success; however, we note that rare variants were annotated by VEP but were not included in the GWAS due to our MAF filter of 0.05 (Figure S12). The 97 genes containing high‐impact variants were significantly over‐represented in a GO term relating to protein binding (GO:0005515) in the GO‐Slim Molecular annotation dataset (Table S6).

**TABLE 1:** Predicted impact of coding region variants across the hihi genome on gene transcripts and protein sequence, as determined by Ensembl VEP. The number and proportion of coding region SNPs in each impact class and variant type are given, with the proportion given in brackets. A total of 258 SNPs were classified as high‐impact due to changes to the start and stop codons.

Impact	Frequency (proportion)	Type	Frequency (proportion)
High	258 (0.87%)	Start lost	46 (0.16%)
		Stop lost	87 (0.29%)
		Stop gained	125 (0.42%)
Moderate	12,076 (40.54%)	Missense variant	12,076 (40.54%)
Low	17,451 (58.59%)	Stop retained variant	9 (0.03%)
		Splice region variant & synonymous variant	390 (1.31%)
		Synonymous variant	17,052 (57.25%)

### 3.3 Inbreeding Depression

**FIGURE 3:**
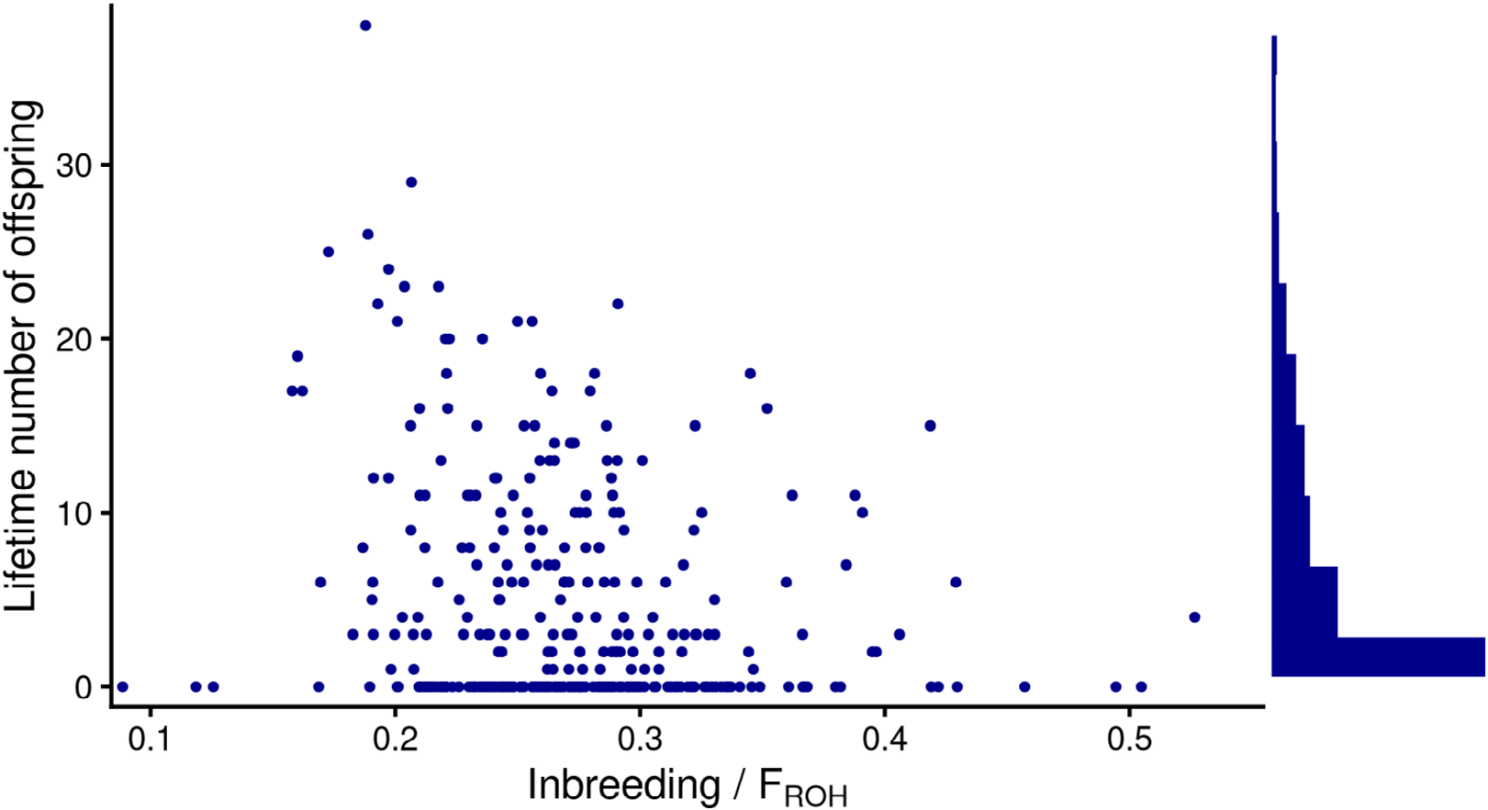
Inbreeding depression in 347 hihi/stitchbird individuals from Tiritiri Matangi. F_ROH_ is the proportion of the genome in ROH—higher F_ROH_ reflects higher inbreeding levels. The scatterplot shows a negative relationship between lifetime reproductive success and inbreeding (F_ROH_). The histogram along the vertical axis shows the distribution of total lifetime number of offspring per individual in the population (mean = 4.93, median = 2, SD = 6.64).

Lifetime reproductive success is uneven across the Tiritiri Matangi population with 42.9% of individuals having no offspring (mean = 4.93, median = 2, SD = 6.64; Figure 3). Individuals with higher F_ROH_ tend to have a lower lifetime number of offspring (Figure 3).

Lifetime reproductive success was modelled with eight metrics that reflect either genome‐wide homozygosity, realised genetic load or inbreeding via runs of homozygosity (see Methods: Modelling inbreeding depression). All measures of homozygosity and inbreeding used in the modelling are significantly correlated with one another (Figure S13), and their count components (non‐zero‐inflated) are highly significant in predicting lifetime reproductive success, except F_ROH>1Mb_, which was only slightly significant (Tables 2 and S7–14). Some, but not all, of the zero‐inflated components of the homozygosity/inbreeding measures were also significant. Sex was not a significant predictor, and the inclusion of an interaction between sex and inbreeding did not improve model fit (Tables S7–14); therefore, we focus here on the non‐interaction models (Table 2). The deviance information criterion (DIC) values reflect goodness of fit to the data, where differences in DIC of > 3 indicate meaningful differences among the models (Spiegelhalter et al. 2002). The proportion of homozygous SNPs, the number of SNPs homozygous for derived alleles, and the number of SNPs homozygous for derived alleles among low‐impact SNPs were the fixed effects that produced the best‐fitting models (DIC ~1383). The number of SNPs homozygous for derived alleles of moderate‐impact SNPs and F_ROH all_ produced models of moderate fit in comparison (DIC ~1386). The number of SNPs homozygous for derived alleles of high‐impact SNPs, F_ROH>300kb_ and F_ROH>1Mb_, produced models of the poorest fit (DIC ~1389). We note that the measures of the number of SNPs homozygous for derived alleles for high‐impact SNPs and F_ROH>1Mb_ represent only a small proportion of the genome in comparison to the number of sites represented in the other measures.

**TABLE 2:** Modelling using MCMCglmm reveals that genome‐wide homozygosity metrics best predict lifetime reproductive success in the hihi/stitchbird. We tested eight metrics representing genome‐wide homozygosity, genetic load and inbreeding (F_ROH_). Models were run with each of the eight metrics, together with sex, as fixed effects. Maternal ID and cohort were included as random effects. All models were run with a zero‐inflated Poisson error distribution and assessed via their deviance information criteria (DIC). All metrics are significantly associated with lifetime reproductive success, even the count of derived alleles for moderate‐ and low‐impact SNPs. Models using genome‐wide homozygosity metrics have the lowest DIC values which indicate the best fit. The posterior mean, credible intervals (CIs) and model support (pMCMC) relate to the count component (non‐zero inflated) of the homozygosity term in the model (***pMCMC < 0.001, *pMCMC < 0.05). Refer to Tables S7–14 for detailed model results, including random effects and zero‐inflated components.

Homozygosity measure	DIC (rank from lowest)	Posterior mean	Lower 95% CI	Upper 95% CI	pMCMC
Genome‐wide homozygosity	1382.566 (1)	−8.9784	−11.8528	−6.0673	< 2e‐05***
Genome‐wide homozygosity at derived sites	1383.637 (2)	−8.80E‐06	−1.17E‐05	−5.81E‐06	< 2e‐05***
Homozogosity at derived high‐impact sites	1390.112 (8)	−0.038	−0.057	−0.020	< 2e‐05***
Homozogosity at derived moderate‐impact sites	1385.478 (4)	−1.70E‐03	−2.30E‐03	−1.09E‐03	< 2e‐05***
Homozogosity at derived low‐impact sites	1383.704 (3)	−1.08E‐03	−1.46E‐03	−6.98E‐04	< 2e‐05***
F_ROH_ – all	1386.329 (5)	−5.3475	−7.7086	−2.904	0.00008***
F_ROH>300kb_	1388.943 (6)	−4.331	−6.441	−2.227	< 2e‐05***
F_ROH>1Mb_	1390.015 (7)	−3.4136	−6.0122	−0.7869	0.0108*

## 4 Discussion

### 4.1 Inbreeding Reflects Population History

The relatively high levels of inbreeding found in the Tiritiri Matangi population reflect the population history of the hihi, and echo our previous genomic inbreeding estimates using a smaller panel of 41,195 markers (Duntsch et al. 2023). Tiritiri Matangi was founded in 1995 by a small number of birds translocated from the remnant population of Te Hauturu‐o‐Toi—40 birds in the first year and 13 birds in the following year (Hihi Recovery Group 2024; Miskelly and Powlesland 2013). Of these 53 birds (28 females, 25 males), only 5 females had offspring, and only 9 males were recorded as social fathers according to available pedigree information (Brekke et al. 2013). The high levels of inbreeding corroborate the small number of founders that actually contributed offspring to later generations, few opportunities for genetic exchange into Tiritiri Matangi over the past 30 years (Nichols et al. 2024), and the small population size (average of ~80 females and ~198 mature individuals over the past 10 years). In comparison, F_ROH_ of other threatened Aotearoa birds are 0.248 in the shore plover ( *Thinornis novaeseelandiae* ; Janes 2023), ~0.1 in the kea ( *Nestor notabilis* ; Stubbs 2022) and 0.02–0.57 in the Rakiura Stewart Island population of kākāpō ( *Strigops habroptilus* ; Dussex et al. 2021; Foster et al. 2021, 2026).

Hihi tend to form social pairs with birds that are more closely related than on average (Brekke et al. 2012), and it is thought that high extra‐pair paternity (EPP) helps reduce overall offspring inbreeding relative to offspring of related social partners (Brekke et al. 2012; Brouwer and Griffith 2019; Castro et al. 2004; Ewen et al. 2004). Our results support previous findings, as we found evidence of first‐degree relative matings that produced highly inbred individuals within the population. Notably, the two most inbred individuals were the result of a social partnership between a mother and her son. However, we also identified individuals with very low inbreeding levels (< 0.2). The Tiritiri Matangi population is male‐biased, and the high rate of EPP may also somewhat attenuate even higher inbreeding levels in the hihi by enabling unpaired, ‘floater’ males to reproduce (Brekke et al. 2015). The attenuation of variance in reproductive success via EPP is also supported by our current and previous results (Duntsch et al. 2023) that indicate no significant effect of sex on fitness, nor any interaction between sex and inbreeding depression, despite its evidence in other wild populations (Vega‐Trejo et al. 2022).

### 4.2 ROH Is Dependent on Input Datasets and Recombination Rates

Despite the use of different SNP densities, input data type (hard calls vs. imputed genotype probabilities) and samples when compared to our previous study (Duntsch et al. 2023) we found very similar F_ROH_ values, and a similar level of variation in inbreeding between individuals in the Tiritiri Matangi population (Figure S8a). The same individuals also showed up as highly inbred (F_ROH_ > 0.5) in both datasets. Looking at the relative distribution of ROH lengths, however, we see that this study with a higher SNP density returns shorter ROH (Figure S8b). Our findings corroborate Duntsch et al. (2021) and Foster et al. (2026) which tested inbreeding estimates across low‐coverage whole genome resequencing, RAD‐seq and/or SNP array densities and found contrasting homozygosity landscapes despite comparable F_ROH_ levels. Both studies also found that both under‐ and over‐estimation of ROH length, as well as differences in ROH presence, contribute to differences in ROH landscape when using different SNP densities (Duntsch et al. 2021; Foster et al. 2026). Lavanchy and Goudet (2023) also found that high‐density SNP sets tend to overestimate short ROHs. A previous study on the hihi found high concordance between genotypes from SNP‐chip and imputed low‐coverage data, which suggests that the differences in ROH results between studies are likely due to different SNP densities instead of imputation (Vi et al. 2025). It is possible that F_ROH_ is most affected by long ROH, which are consistently detected even at lower SNP densities, particularly in small inbred populations (Lavanchy and Goudet 2023). Longer ROHs could also be broken into adjacent shorter ROHs due to previously untyped heterozygous genotypes. F_ROH_ could therefore be less sensitive to SNP densities, while positions and lengths of ROH are more sensitive, which contributes to discordance in homozygosity landscapes across different datasets. The interpretation of HBD classes in the context of coalescent time should therefore be considered in relation to the input dataset and the parameters used.

The significant correlation between recombination rates and ROH density has been shown in other studies where ROH length and position are inversely correlated with local recombination rates (Ceballos et al. 2018; Pemberton et al. 2012; Stoffel et al. 2021a). Recombination breaks down long haplotypes, which results in shorter ROH and a decrease in the power of detecting ROH, hence leading to lower ROH density (Hewett et al. 2023; Kardos et al. 2017). However, albeit significant, the correlation between recombination and ROH density is weak, and therefore ROH reflects the contribution of parental relatedness, population history and selection, on top of just the lack of recombination (Hewett et al. 2023; Pemberton et al. 2012).

### 4.3 Differences in Genetic Load Results Across Methods

Genetic load, quantified in our GWAS using direct fitness measures, returned only a handful of highly significant regions. In our GWAS, we applied Bonferroni correction, which assumes that SNPs represent independent tests, which is a stringent threshold as linkage disequilibrium exists for SNPs in close proximity (Fadista et al. 2016; Lee et al. 2022). Given our modest sample size, we adopted conservative thresholds to minimise the risk of false positives by removing SNPs with fewer than 5 observations in the rarest genotype class. However, this likely resulted in the exclusion of a few significant SNPs that may represent true associations. In contrast to the small number of associated regions from our GWAS, genetic load quantified using predicted functional impacts identified many high‐impact SNPs that affect gene start and stop codons (Figure S12). One reason for the low concordance between the high‐impact SNPs and our GWAS results is that some of these high‐impact SNPs had low MAF and were filtered out prior to GWAS. Another possible explanation is that within coding regions, purging has been successful in removing some of the most impactful variants, and those remaining are close to being lost from the population. The challenges of accurately detecting and analysing low‐frequency or rare variants in GWAS are known limitations (Bansal et al. 2010; Lee et al. 2014), especially when rare variants have been shown to significantly contribute to common diseases in humans (Wang et al. 2021). There was no overlap in GO enrichment terms between our GWAS and genes with high‐impact SNPs, but we were able to corroborate our GWAS findings with those of other studies by identifying genes related to reproductive traits and key developmental pathways, which contribute to our understanding of reproductive success in birds. Future work employing a larger number of individuals and high‐coverage sequences could further investigate the associations between lifetime reproductive success and the regions of interest identified in this study, and evaluate the impacts of imputation and filtering on false negatives.

### 4.4 Building a More Complete Understanding of Inbreeding Depression in Hihi

Our results add to previous studies that tested for inbreeding depression in the Tiritiri Matangi population of hihi (Brekke et al. 2010; Duntsch et al. 2023; Morland et al. 2024a; Nichols et al. 2024; Stuart et al. 2024). Notably, this study represents the most comprehensive, genome‐scale dataset analysed to date and provides strong evidence for a reduction in lifetime reproductive success with increased inbreeding levels, whether measured by genome‐wide homozygosity, within‐gene homozygosity or runs of homozygosity.

Brekke et al. (2010) found that inbreeding, as measured from 19 microsatellite markers, had an impact on embryo and nestling survival in a single breeding season. Microsatellite‐based inbreeding measures are limited by the low number of markers and may not correlate strongly with genome‐wide inbreeding, as supported by the observation that heterozygosity calculated from a relatively large dataset of 18,415 RADseq markers is poorly correlated (~0.20) with genome‐wide heterozygosity from 644,631 high‐confidence SNPs (Table S9 in Duntsch et al. (2020)). In contrast, using inbreeding calculated from available pedigree data informed by microsatellites, Nichols et al. (2024) found no significant effect of inbreeding on hatching, fledging or recruitment, and Morland et al. (2024a) found no significant effect of inbreeding on early embryo death. Many studies have demonstrated that genetic markers, especially at higher densities, are more precise in estimating realised inbreeding than that from pedigrees (Kardos et al. 2015, 2016; Wang 2016).

Our first genomic study of inbreeding depression utilised F_ROH_ calculated from 41,195 SNPs, and used lifetime reproductive success after adjusting for lifespan as a measure of fitness (Duntsch et al. 2023). The study found no significant relationship with genome‐wide inbreeding but detected 13 SNPs in 3 regions of the genome where ROH status was significantly negatively associated with lifespan‐adjusted reproductive success. In contrast we tested lifetime reproductive success directly in this study and detected inbreeding depression at the genome level, identifying different genomic regions associated with fitness. Given that different traits were tested (lifespan‐adjusted versus lifespan‐unadjusted reproductive success), the differences in results are unsurprising. Here, we chose not to adjust for lifespan to give a more complete fitness measure (Figure S14). Further, although individuals largely overlapped between the studies, the SNP datasets were of substantially different magnitude—Duntsch et al. (2023) employed 363 individuals genotyped on a SNP chip, whereas we have 347 individuals sequenced and imputed to 2,329,893 genotyped SNPs. Comparisons of SNP chip versus imputed low‐coverage whole‐genome sequencing datasets of pigs found improvements in the accuracy of genomic prediction and identification of associated genes in the latter (Wang et al. 2024; Z. Zhang, et al. 2022). The differences in region‐specific associations may also be related to the differences in ROH lengths between datasets, which results in differing statistical power in identifying significant associations (Stoffel et al. 2021b).

We also note a difference in our GWAS approach compared to Duntsch et al. (2023)—our previous study followed the approach of Stoffel et al. (2021a) to test trait association with a SNP allele that is also in an ROH, thereby capturing linkage disequilibrium with untyped homozygous variants. In this study, we have (almost) whole‐genome SNP variants and so should have typed the vast majority of causative SNP variants, including ones in strong linkage disequilibrium with non‐SNP variants (Stuart et al. 2024). Thus, we used a dominance GWAS model to capture association with causal variants that contribute to inbreeding depression via either increased homozygosity for recessive deleterious alleles or increased homozygosity for alleles with heterozygote advantage.

Finally, our results are concordant with our previous study which demonstrated that both realised SNP load and realised structural variant (SV) load predicted fitness (lifetime reproductive success) in 28 successful hihi breeders (Stuart et al. 2024). In that analysis, it was assumed that rare variants were more deleterious, and load was quantified by summing the number of homozygous rare alleles per individual for both SNPs and SVs. Here, we leverage inference of an ancestral passerine genome to polarise our SNP alleles and therefore quantify genetic load as homozygosity at derived high‐impact alleles. This approach is more concordant with the approach of other studies quantifying load (Cavill et al. 2024; Smeds and Ellegren 2023), and recognises that deleterious variants may not necessarily be at low frequency in the population due to the small population size and therefore the considerable impact of genetic drift (Robinson et al. 2023).

In summary, our genome‐scale analyses of a much larger number of individuals, including a substantial proportion that did not breed, provide support for the hypothesis that inbreeding affects survival and reproduction throughout the entire lifespan of individuals in the Tiritiri Matangi population. Future studies will focus on the impact of genomic inbreeding on other populations (e.g., the Zealandia population, which has lower diversity; Brekke et al. 2011) and on its impact on the ‘missing fraction’ represented by early embryo mortality (Morland et al. 2024b).

### 4.5 Direct Measures of Fitness and Genome‐Wide Homozygosity Improve Detection of Inbreeding Depression

We found genetic load predicted from variant effect predictor analyses to be a misleading proxy for inbreeding depression. Our results corroborate previous studies which found that genetic load is not the best predictor of fitness (Hoelzel et al. 2024; Robledo‐Ruiz et al. 2025). Genetic load measured from the count of derived homozygous alleles is, however, correlated with genome‐wide homozygosity. Therefore, the significant relationship between the count of derived homozygous alleles of high‐impact SNPs and fitness is likely a reflection of the relationship between fitness and genome‐wide homozygosity, since the signal is also similar in moderate‐ and low‐impact SNPs. Our hypothesis is further backed up by the observation that high‐impact SNPs have poorer power in predicting fitness than moderate‐ and low‐impact SNPs as they represent a much smaller fraction of the genome. This suggests that being homozygous for many 'high‐impact' genetic variants does not necessarily reflect a negative fitness outcome. While genetic load measures changes at the amino acid/protein level, these changes may not translate into actual impact on fitness. On the other hand, direct measures of fitness operate at the individual level, and in the context of interactions with the environment and other species, and are therefore invaluable for assessing not only the impacts of inbreeding on fitness, but also other key contributors such as sex and age. Our findings agree with recent work by Robledo‐Ruiz et al. (2025), which found that F_ROH_ was a better predictor for fitness than realised load measured from genome conservation scores, which are often inconsistent. A study by Hoelzel et al. (2024) on the northern elephant seal also found that both the number of pups weaned annually and over a female's lifetime are more correlated with F_ROH_ than the number of loss‐of‐function alleles with homozygous genotypes. While mutation load remains an important aspect to consider in cases of potential outbreeding depression (Dussex et al. 2023), more evaluations of its effectiveness are needed for non‐model organisms, and we recommend the use of direct measures of fitness where possible.

In our study, genome‐wide homozygosity was the best predictor of fitness. Previous studies on this correlation are split, with some studies finding strong correlations (Harrisson et al. 2019; Hoffman et al. 2014) while not in others (Chapman and Sheldon 2011; Rodríguez‐Quilón et al. 2015). The differences in outcomes could be attributable to differences in input data—different measures of fitness and different marker densities that affect the accuracy of genome‐wide homozygosity estimates (Hansson and Westerberg 2008)—and inherent differences in population genetic histories or genome architecture, such as recombination rates. Homozygosity‐fitness correlations will also only be apparent when there is sufficient variance in inbreeding, and so will not be detected in populations where all individuals are near equally inbred (Slate et al. 2004).

Where the genetic effects of inbreeding on fitness were once debated, we now have many studies on both wild (Hasselgren et al. 2021; Hewett et al. 2024; Hewett et al. 2025a; Niskanen et al. 2020; Stoffel et al. 2021a) and captive animal populations (Ablondi et al. 2023; Bem et al. 2024; Chu et al. 2019; Y. Zhang et al. 2022) that have demonstrated a negative and significant association between F_ROH_ and fitness, as measured by survival or reproductive phenotypic traits. In these studies and ours, we demonstrate that F_ROH_ is a significant predictor of fitness and is valuable for identifying populations at risk and genomic monitoring of inbred populations with the goal of conservation management (Kyriazis et al. 2025a). The finding that models using F_ROH_, although still significant, had a slightly poorer fit than both genome‐wide homozygosity and genetic load measures was surprising. This finding is also slightly counter to recent work by Robledo‐Ruiz et al. (2025), which found that F_ROH_ was a better predictor for fitness than genome‐wide homozygosity. The utility of F_ROH_ and genome‐wide homozygosity measures in predicting fitness requires further investigation (Shafer and Kardos 2025). F_ROH_ may have lower statistical power, especially when high cutoffs of ROH are applied, but could also be useful in datasets of lower SNP density where ROH can bridge genomic regions containing untyped SNPs. Additionally, the idea that genome‐wide homozygosity captures the effects of structural variants, and therefore unique aspects of genetic variation and load (Stuart et al. 2024), better than ROH, warrants further exploration.

## 5 Summary

We found high levels of inbreeding in the Tiritiri Matangi population of hihi, which is negatively associated with lifetime reproductive success, suggesting inbreeding depression. Inbreeding levels in the hihi reflect the population history of bottlenecks and isolation in small populations, corroborating past studies. Recent inbreeding events involving close relatives result in highly inbred individuals, but high extra‐pair paternity in the hihi may somewhat mitigate the consequences of close inbreeding in social pairs. In addition to parental relatedness, ROH density is also associated with recombination landscapes across the genome. We identified several regions in the genome associated with lifetime reproductive success that harbour genes linked to reproduction in other birds. While we found many moderate‐ and high‐impact SNPs predicted to change translated amino acid sequences, their association with fitness data is likely to reflect the genome‐wide impact of homozygosity on fitness. Our finding of inbreeding depression in the hihi provides support for genetic exchange to reduce inbreeding levels and achieve positive conservation outcomes that may improve the adaptive potential of the hihi (Brekke et al. 2011; Frankham 2015; Hedrick and Garcia‐Dorado 2016). Our work provides a workflow for researchers hoping to use imputation on low‐coverage whole‐genome resequencing to characterise inbreeding depression in their study species.

## Funding

A Strategic Science Investment Fund in Data Science from the New Zealand Ministry of Business, Innovation and Employment (MBIE) supports Anna W. Santure and Hui Zhen Tan. The MBIE Genomics Aotearoa High Quality Genomes and High Quality Genomes and Population Genomics (HQG + PG) projects I and II supported Anna W. Santure and Katarina C. Stuart and funded the whole‐genome resequencing data and sequencing of the reference genome utilised in this study. Funding from the New Zealand George Mason Center for the Natural Environment and the Little Barrier Island (Hauturu) Supporters Trust supported fieldwork collection from Te Hauturu‐o‐Toi. Tram Vi was supported by a University of Auckland Faculty of Science Research Development Fund awarded to Anna W. Santure. Patricia Brekke is supported by Research England.

## Conflicts of Interest

The authors declare no conflicts of interest.

## Supporting information


**Figure S1:** Details of population sampling of hihi/stitchbird. Map on the left depicts the North Island of Aotearoa, New Zealand, and the points show the location of present‐day hihi populations. Populations that were sampled in our study are in dark blue (1: Te Hauturu‐o‐Toi, 2: Tiritiri Matangi, 3: Zealandia Te Māra a Tāne Wildlife Sanctuary), while all other populations are in light blue. Arrows on the map indicate the translocation history and genealogical relationships among the three sampled populations: birds from Te Hauturu‐o‐Toi (remnant population) served as founders of Tiritiri Matangi, which in turn provided birds that formed two‐thirds of the founders for Zealandia, alongside birds from a captive population established by birds from Te Hauturu‐o‐Toi. After sample checks, a total of 30 individuals formed the imputation reference panel, while 401 verified Tiritiri Matangi samples were retained as the target for imputation.
**Figure S2:** Box plot of per‐individual heterozygosity values. Heterozygosity was calculated as the proportion of heterozygous SNPs (mean = 0.148, SD = 0.05). The red dot represents the individual that was removed due to excess heterozygosity.
**Figure S3:** Comparison of genetic versus pedigree relatedness pre‐ and post‐sample checks. Genetic relatedness versus pedigree relatedness for 410 Tiritiri Matangi birds before (grey points) and after (black points) sample checks, where only verified individuals were retained. Focal clusters for our checks include (a) parent–child relationships, (b) half‐sib/grandparent–grandchild/uncle/aunt to nephew/niece relationships and (c) unrelated pairs.
**Figure S4:** Sample checks reveal multiple discordant pairwise relationships for two problematic samples. Plots of pedigree versus genetic relatedness pre‐sample checks reveal many erroneous pairwise relationships (red points) involving two problematic samples, in contrast to the pairwise relationships across all other individuals (grey points). Both samples had poor sequencing quality and were removed from downstream analyses.
**Figure S5:** Sample checks reveal eight samples with disagreement between recorded sex and genetic sex. Recorded sex is indicated by the colour of the bars, while genetic sex is indicated by the proportion of heterozygosity in the Z chromosome. Male birds, which are homogametic (ZZ), are expected to have higher heterozygosity than female birds, which are heterogametic (ZW). Of the eight samples (indicated by asterisks) identified as showing disagreement between recorded and genetic sex, four were also problematic in relatedness checks and were removed from downstream analyses. The remaining four samples were verified in relatedness checks and likely to indicate errors in recorded sex due to misidentifications in the field.
**Figure S6:** No batch effects were detected through principal component analysis (PCA) of SNPs pre‐imputation. In the PCAs, samples show differentiation by population (top panel), but not by sequencing batch (bottom panel).
**Figure S7:** Distribution of imputation accuracy values, as measured by Beagle Dosage R‐Squared (DR2), for bins of folded minor allele frequency (MAF). SNPs generally have high DR2 values, but SNPs with low MAF exhibit lower median DR2 and increased variation. The histogram on the right represents the distribution of DR2 values across all SNPs. SNPs with DR2 values lower than 0.9 were excluded from all analyses.
**Figure S8:** Comparison of RZooRoH results between this study and Duntsch et al. (2023). We compared (a) F_ROH>300kb_ and (b) mean ROH length for 268 individuals that were shared by both studies. In both plots, each point represents an individual, and the red dashed line represents a 1:1 correlation. Correlations between studies were tested for each value respectively using Kendall's test (non‐parametric). Both measurements were significantly correlated between the two studies, but Duntsch et al. (2023) found longer ROHs on average.
**Figure S9:** ROH density versus sex‐averaged recombination rate across the hihi genome. Points on the figure represent non‐overlapping 500 kb intervals. ROH density refers to the number of individuals in ROH in each interval. Kendall's test (non‐parametric) revealed significant, negative correlations between ROH density and sex‐averaged recombination rate. The solid grey line represents the linear regression, and the shaded area represents the 95% confidence interval.
**Figure S10:** Genome‐wide association study (GWAS) of SNP effects on the lifetime reproductive success of 347 hihi/stitchbird individuals from Tiritiri Matangi using the additive model. Lifetime reproductive success is the total number of offspring an individual has over its lifetime and was only calculated for individuals who have completed their reproductive lifespan. The red dashed line represents the Bonferroni‐corrected significance threshold (−log10(p) > 7.62). Axis ticks after chromosome 21 represent chromosomes 22, 23, 24, 25A, 25B, 26 (labelled in figure), 27, 28, 29.
**Figure S11:** Box plots of derived allele frequency of SNPs in respective Variant Effect Predictor (VEP) impact classes. High‐impact SNPs have the lowest median derived allele frequency, followed by moderate‐ then low‐impact SNPs.
**Figure S12:** Dominant GWAS with high‐impact SNPs. SNPs in blue represent the results of each SNP analysed in the TASSEL GWAS, while SNPs in red represent high‐impact SNPs, predicted from Ensembl VEP, that were analysed in the GWAS. SNPs in green (at the top of the plot) were not included in the GWAS due to the applied MAF filter of 0.05, that is, log likelihoods of green points were not calculated but are in a line at the top of the plot for visualisation purposes. We note a high‐impact SNP (predicted as a stop gain/loss variant in the gene ATP2C1) close to the significant SNP on chromosome 2 (10,043 bp away). It was included in the GWAS analysis but subsequently removed from visualisation because it had only one individual with the rare homozygote genotype AA. Genotype effects from the dominance model were very similar for all genotype classes AA (1 individual), AC (251 individuals) and CC (95 individuals), and the *p*‐value on the model was 0.701. Therefore, there was no evidence that this predicted high‐impact SNP is causative.
**Figure S13:** Correlation between different measures of homozygosity, genetic load and inbreeding. Correlation results between two genome‐wide homozygosity measures (proportion of homozygous SNPS, derived homozygosity count), 3 genetic load measures (derived homozygous counts of high‐, moderate‐ (‘mod‐’) and low‐impact SNPs) and 3 F_ROH_ measures (F_ROH all_, F_ROH>300kb_, F_ROH>1Mb_), as labelled at the top of the figure. Panels above the diagonal show correlation values; all correlations are highly significant *p* < 0.001, as indicated by ***. Density plots are on the diagonal, while plots below the diagonal show scatterplots. Labels on the right edge of the plot correspond to labels at the top of the figures.
**Figure S14:** Scatterplot of lifetime number of offspring against longevity (in days) of 347 hihi/stitchbird individuals from Tiritiri Matangi. The total number of offspring per individual was only calculated for individuals who had completed their reproductive lifespan. The histogram along the vertical axis shows the distribution of longevity in the population.
**Table S1:** Details of VEP input format for different SNP categories. We divided our SNPs into four categories based on the inferred ancestral allele. We present here the proportion of SNPs in each category, along with the format used for input into VEP (AA = ancestral allele, REF = original reference allele, ALT = original alternate alleles).
**Table S2:** TASSEL results for the two most significant SNPs on chromosome 2 at 41,987,466 bp and on chromosome 3 at 70,449,698 bp. The ‘Effect’ and ‘Obs’ columns represent the estimated effects of each genotype and the number of observations from the dataset. The remaining columns show the model statistics for the dominance model for each SNP.
**Table S3:** MCMCglmm results using genotype at 41,987,466 bp on chromosome 2 as a fixed effect for lifetime reproductive success (LRS). The model was run using a zero‐inflated Poisson error distribution with SNP genotype and sex as fixed factors. Maternal ID (dam_ID), cohort and genetic relatedness (animal) were included as random effects. The model and resulting deviance information criterion (DIC) are provided in the first row. ‘traitLRS’ indicates the count component (non‐zero‐inflated) of the respective term, while ‘traitzi_LRS’ indicates the zero‐inflated component. The first two terms represent the model intercept.
**Table S4:** MCMCglmm results using genotype at 70,449,698 bp on chromosome 3 as a fixed effect for lifetime reproductive success (LRS). The model was run using a zero‐inflated Poisson error distribution with SNP genotype and sex as fixed factors. Maternal ID (dam_ID), cohort and genetic relatedness (animal) were included as random effects. The model and resulting deviance information criterion (DIC) are provided in the first row. ‘traitLRS’ indicates the count component (non‐zero‐inflated) of the respective term, while ‘traitzi_LRS’ indicates the zero‐inflated component. The first two terms represent the model intercept.
**Table S5:** Gene ontology (GO) terms overrepresented in 298 genes containing 1460 strongly associated SNPs (*p* < 0.0001) from our GWAS with the dominant model.
**Table S6:** GO terms overrepresented in 97 genes containing 258 SNPs predicted by Ensembl VEP to be high‐impact.
**Table S7:** MCMCglmm results using genome‐wide proportion of homozygous SNPs (‘hom_prop’) as a fixed effect for lifetime reproductive success (LRS). All models (Tables S7–14) were run using a zero‐inflated Poisson error distribution with a metric of homozygosity/genetic load/inbreeding, respectively, as well as sex as fixed effects. Maternal ID (dam_ID) and cohort were included as random effects. The model and resulting deviance information criterion (DIC) are provided in the first row. ‘traitLRS’ indicates the count component (non‐zero‐inflated) of the respective term, while ‘traitzi_LRS’ indicates the zero‐inflated component. The first two terms represent the model intercept.
**Table S8:** MCMCglmm results using genome‐wide count of homozygous derived alleles (‘hom_derived_count’) as a fixed effect for lifetime reproductive success (LRS). All models (Tables S7–14) were run using a zero‐inflated Poisson error distribution with a metric of homozygosity/genetic load/inbreeding, respectively, as well as sex as fixed effects. Maternal ID (dam_ID) and cohort were included as random effects. The model and resulting deviance information criterion (DIC) are provided in the first row. ‘traitLRS’ indicates the count component (non‐zero‐inflated) of the respective term, while ‘traitzi_LRS’ indicates the zero‐inflated component. The first two terms represent the model intercept.
**Table S9:** MCMCglmm results using the count of homozygous derived high‐impact SNPs (‘hom_derived_count_high’) as fixed effect for lifetime reproductive success (LRS). All models (Tables S7–14) were run using a zero‐inflated Poisson error distribution with a metric of homozygosity/genetic load/inbreeding, respectively, as well as sex as fixed effects. Maternal ID (dam_ID) and cohort were included as random effects. The model and resulting deviance information criterion (DIC) are provided in the first row. ‘traitLRS’ indicates the count component (non‐zero‐inflated) of the respective term, while ‘traitzi_LRS’ indicates the zero‐inflated component. The first two terms represent the model intercept.
**Table S10:** MCMCglmm results using count of homozygous derived moderate‐impact SNPs (‘hom_derived_count_mod’) as fixed effect for lifetime reproductive success (LRS). All models (Tables S7–14) were run using a zero‐inflated Poisson error distribution with a metric of homozygosity/genetic load/inbreeding, respectively, as well as sex as fixed effects. Maternal ID (dam_ID) and cohort were included as random effects. The model and resulting deviance information criterion (DIC) are provided in the first row. ‘traitLRS’ indicates the count component (non‐zero‐inflated) of the respective term, while ‘traitzi_LRS’ indicates the zero‐inflated component. The first two terms represent the model intercept.
**Table S11:** MCMCglmm results using count of homozygous derived low‐impact SNPs (‘hom_derived_count_low’) as fixed effect for lifetime reproductive success (LRS). All models (Tables S7–14) were run using a zero‐inflated Poisson error distribution with a metric of homozygosity/genetic load/inbreeding, respectively, as well as sex as fixed effects. Maternal ID (dam_ID) and cohort were included as random effects. The model and resulting deviance information criterion (DIC) are provided in the first row. ‘traitLRS’ indicates the count component (non‐zero‐inflated) of the respective term, while ‘traitzi_LRS’ indicates the zero‐inflated component. The first two terms represent the model intercept.
**Table S12:** MCMCglmm results using F_ROH all_ (‘froh_all’) as fixed effect for lifetime reproductive success (LRS). All models (Tables S7–14) were run using a zero‐inflated Poisson error distribution with a metric of homozygosity/genetic load/inbreeding, respectively, as well as sex as fixed effects. Maternal ID (dam_ID) and cohort were included as random effects. The model and resulting deviance information criterion (DIC) are provided in the first row. ‘traitLRS’ indicates the count component (non‐zero‐inflated) of the respective term, while ‘traitzi_LRS’ indicates the zero‐inflated component. The first two terms represent the model intercept.
**Table S13:** MCMCglmm results using F_ROH>300kb_ (‘froh_300kb’) as fixed effect for lifetime reproductive success (LRS). All models (Tables S7–14) were run using a zero‐inflated Poisson error distribution with a metric of homozygosity/genetic load/inbreeding, respectively, as well as sex as fixed effects. Maternal ID (dam_ID) and cohort were included as random effects. The model and resulting deviance information criterion (DIC) are provided in the first row. ‘traitLRS’ indicates the count component (non‐zero‐inflated) of the respective term, while ‘traitzi_LRS’ indicates the zero‐inflated component. The first two terms represent the model intercept.
**Table S14:** MCMCglmm results using F_ROH>1Mb_ (‘froh_1Mb’) as fixed effect for lifetime reproductive success (LRS). All models (Tables S7–14) were run using a zero‐inflated Poisson error distribution with a metric of homozygosity/genetic load/inbreeding, respectively, as well as sex as fixed effects. Maternal ID (dam_ID) and cohort were included as random effects. The model and resulting deviance information criterion (DIC) are provided in the first row. ‘traitLRS’ indicates the count component (non‐zero‐inflated) of the respective term, while ‘traitzi_LRS’ indicates the zero‐inflated component. The first two terms represent the model intercept.

## Data Availability

Hihi are of cultural significance to the Indigenous People of Aotearoa New Zealand, the Māori and are considered a taonga (treasured) species whose whakapapa (genealogy) is intricately tied to that of Māori. For this reason, sequencing and variant data are archived on the Aotearoa Genomic Data Repository (AGDR) and can be viewed using the Project Code NZ‐00068 and the Dataset ID AGDR00105 (https://doi.org/10.57748/tz1n‐sn93). These data will be made available by request on the recommendation of Ngāti Manuhiri, the iwi (extended kinship group) that affiliates as kaitiaki (guardians) for hihi. Pipelines and analysis codes are available at https://github.com/tanhuizhen/Hihi_Inbreeding‐depression. Additional data supporting our findings, including information on sample checks and modelling results, are available as Supporting Information.
