# Supplementary material for: Genome‐Wide Homozygosity Predicts Inbreeding Depression in the Hihi/Stitchbird (Notiomystis cincta) Better Than Realised Load
Source: Evol Appl. 2026 May 21;19(5):e70252. doi: 10.1111/eva.70252 (PMC13240288; doi:10.1111/eva.70252)
Supplement: Supplementary file 1 — Figure S1: Details of population sampling of hihi/stitchbird. Map on the left depicts the North Island of Aotearoa, New Zealand, and the points show the location of present‐day hihi populations. Populations that were sampled in our study are in dark blue (1: Te Hauturu‐o‐Toi, 2: Tiritiri Matangi, 3: Zealandia Te Māra a Tāne Wildlife Sanctuary), while all other populations are in light blue. Arrows on the map indicate the translocation history and genealogical relationships among the three sampled populations: birds from Te Hauturu‐o‐Toi (remnant population) served as founders of Tiritiri Matangi, which in turn provided birds that formed two‐thirds of the founders for Zealandia, alongside birds from a captive population established by birds from Te Hauturu‐o‐Toi. After sample checks, a total of 30 individuals formed the imputation reference panel, while 401 verified Tiritiri Matangi samples were retained as the target for imputation. Figure S2: Box plot of per‐individual heterozygosity values. Heterozygosity was calculated as the proportion of heterozygous SNPs (mean = 0.148, SD = 0.05). The red dot represents the individual that was removed due to excess heterozygosity. Figure S3: Comparison of genetic versus pedigree relatedness pre‐ and post‐sample checks. Genetic relatedness versus pedigree relatedness for 410 Tiritiri Matangi birds before (grey points) and after (black points) sample checks, where only verified individuals were retained. Focal clusters for our checks include (a) parent–child relationships, (b) half‐sib/grandparent–grandchild/uncle/aunt to nephew/niece relationships and (c) unrelated pairs. Figure S4: Sample checks reveal multiple discordant pairwise relationships for two problematic samples. Plots of pedigree versus genetic relatedness pre‐sample checks reveal many erroneous pairwise relationships (red points) involving two problematic samples, in contrast to the pairwise relationships across all other individuals (grey points). Both samples had [file EVA-19-e70252-s001.docx]

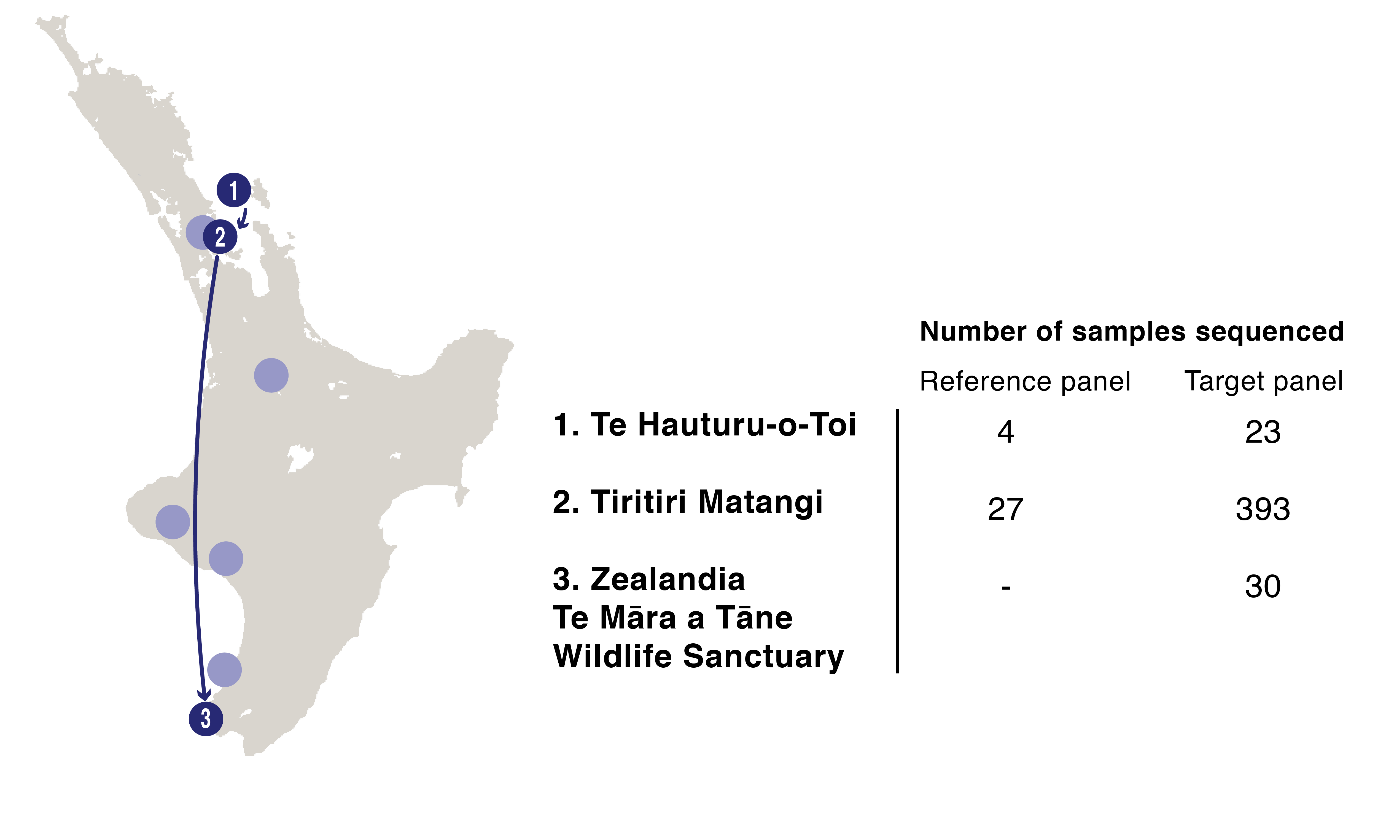


**Figure S1. Details of population sampling of hihi / stitchbird.** Map on the left depicts the North Island of Aotearoa New Zealand and points show the location of present-day hihi populations. Populations that were sampled in our study are in dark blue (1: Te Hauturu-o-Toi, 2: Tiritiri Matangi, 3: Zealandia Te Māra a Tāne Wildlife Sanctuary), while all other populations are in light blue. Arrows on the map indicate the translocation history and genealogical relationships among the three sampled populations: birds from Te Hauturu-o-Toi (remnant population) served as founders of Tiritiri Matangi, which in turn provided birds that formed two-thirds of the founders for Zealandia, alongside birds from a captive population established by birds from Te Hauturu-o-Toi. After sample checks, a total of 30 individuals formed the imputation reference panel, while 401 verified Tiritiri Matangi samples were retained as the target for imputation.


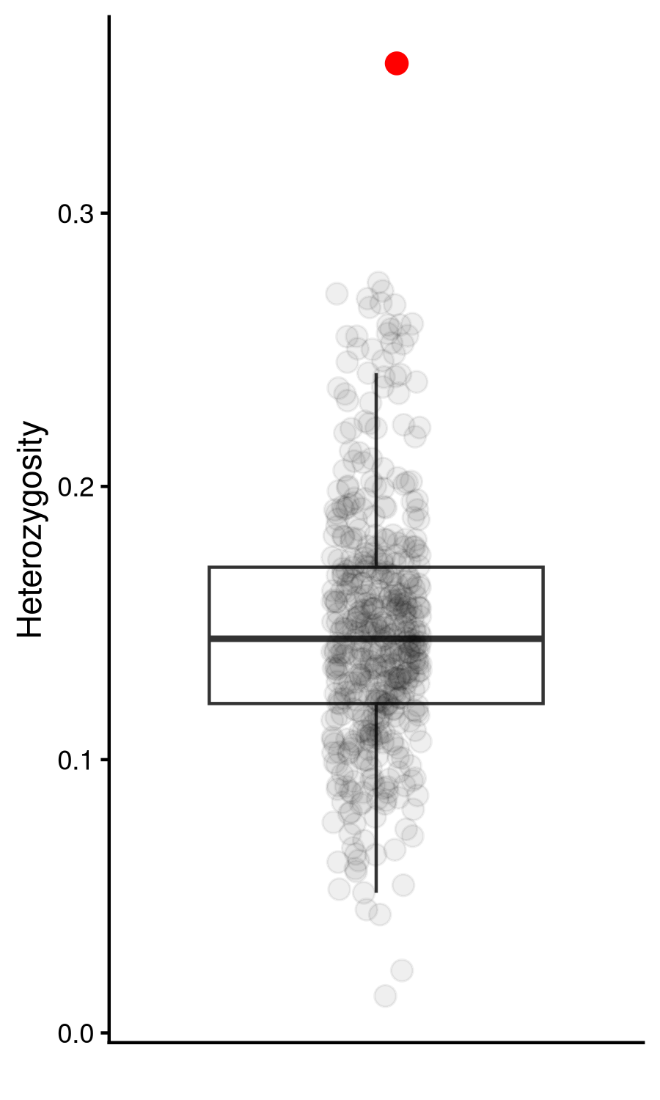


**Figure S2. Box plot of per-individual heterozygosity values.** Heterozygosity was calculated as the proportion of heterozygous SNPs (mean = 0.148, sd = 0.05). The red dot represents the individual that was removed due to excess heterozygosity.


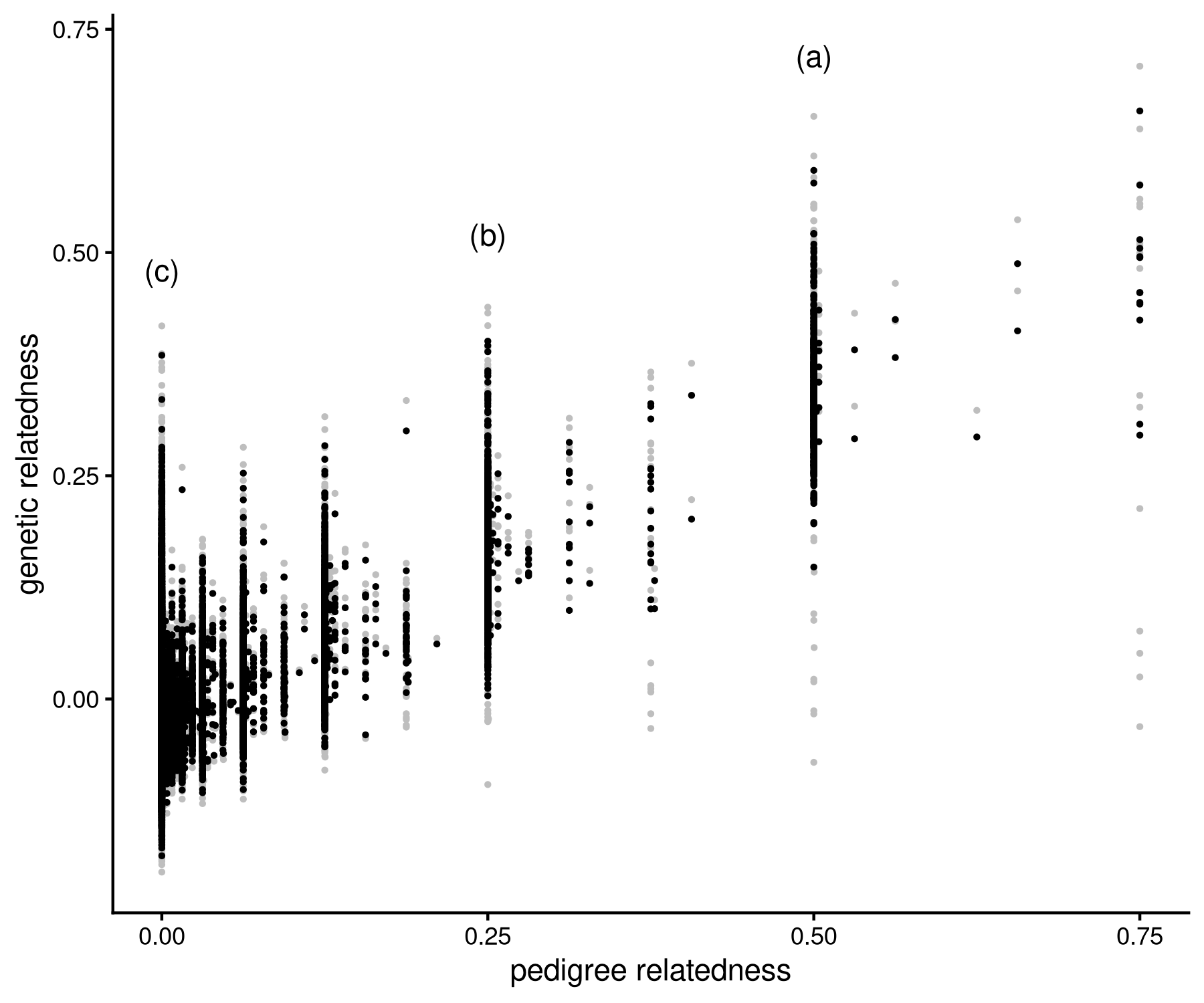


**Figure S3. Comparison of genetic vs pedigree relatedness pre- and post-sample checks.** Genetic relatedness vs pedigree relatedness for 410 Tiritiri Matangi birds before (grey points) and after (black points) sample checks, where only verified individuals were retained. Focal clusters for our checks include (a) parent-child relationships, (b) half-sib / grandparent-grandchild / uncle/aunt to nephew/niece relationships, and (c) unrelated pairs.


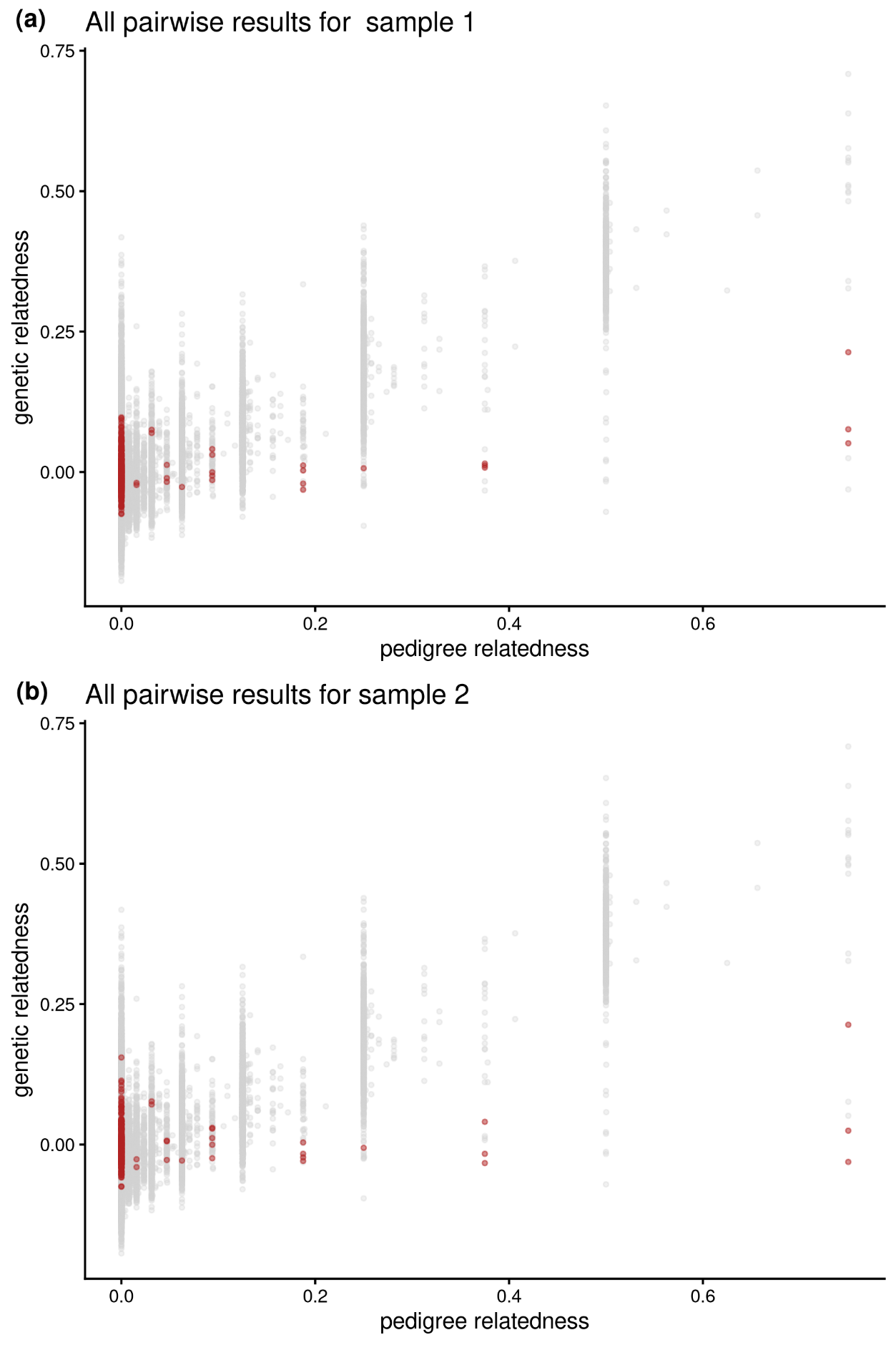


**Figure S4. Sample checks reveal multiple discordant pairwise relationships for two problematic samples.** Plots of pedigree versus genetic relatedness pre-sample checks reveal many erroneous pairwise relationships (red points) involving two problematic samples, in contrast to the pairwise relationships across all other individuals (grey points). Both samples had poor sequencing quality and were removed from downstream analyses.


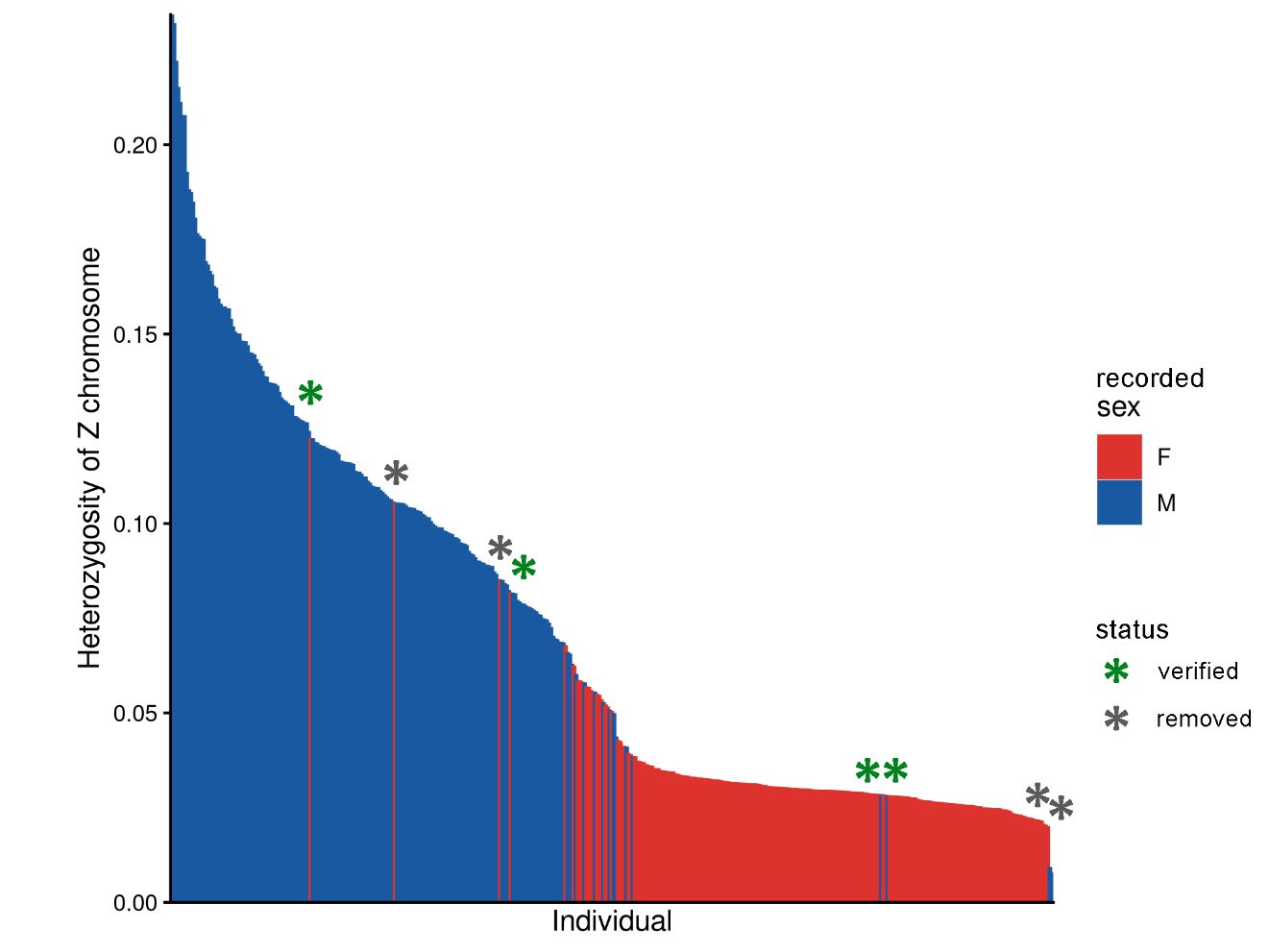


**Figure S5. Sample checks reveal eight samples with disagreement between recorded sex and genetic sex.** Recorded sex is indicated by the colour of the bars while genetic sex is indicated by the proportion of heterozygosity in the Z chromosome. Male birds, which are homogametic (ZZ), are expected to have higher heterozygosity than female birds, which are heterogametic (ZW). Of the eight samples (indicated by asterisks) identified as showing disagreement between recorded and genetic sex, four were also problematic in relatedness checks and were removed from downstream analyses. The remaining four samples were verified in relatedness checks and likely to indicate errors in recorded sex due to misidentifications in the field.


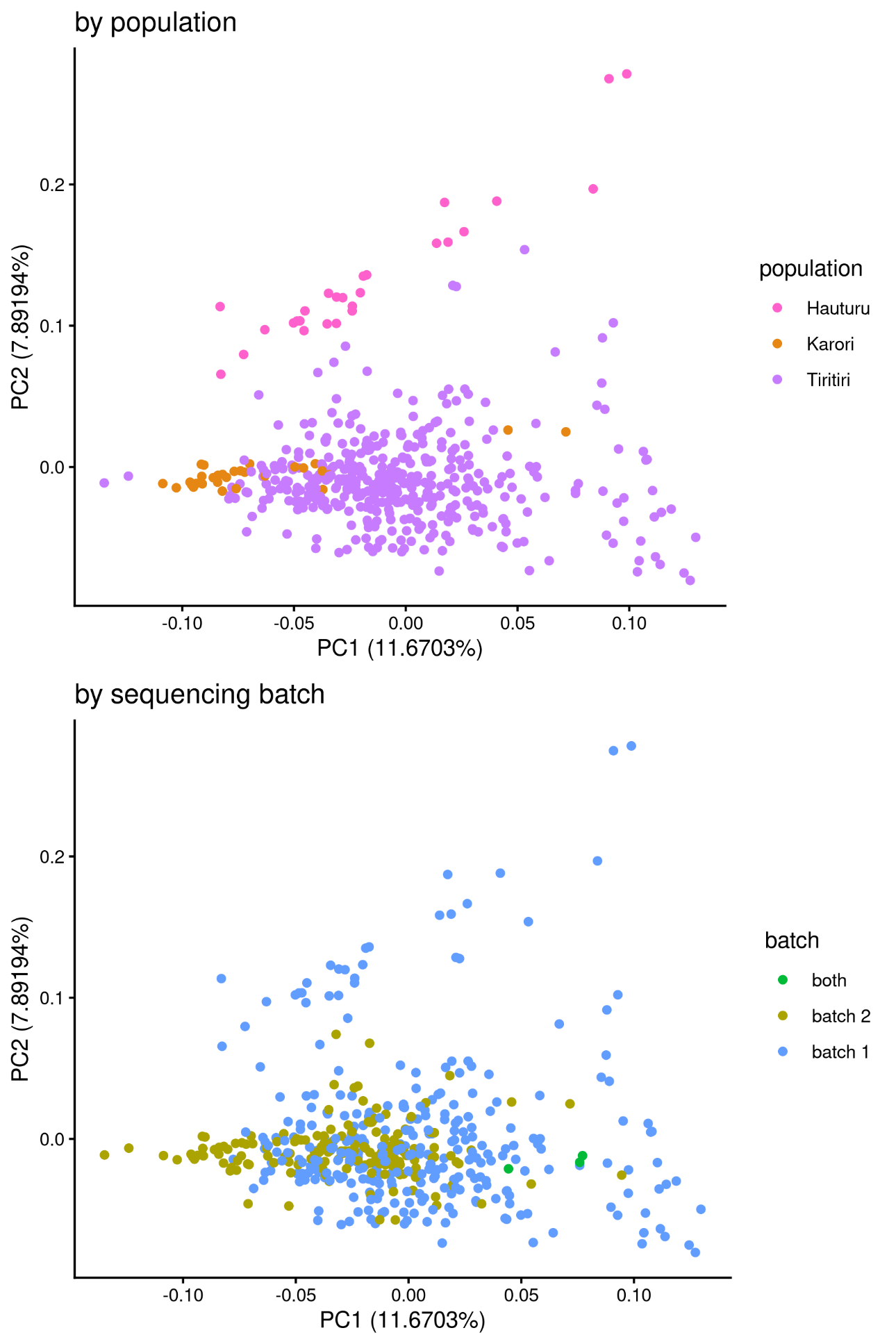


**Figure S6. No batch effects were detected through principal component analysis (PCA) of SNPs pre-imputation.** In the PCAs, samples show differentiation by population (top panel), but not by sequencing batch (bottom panel).
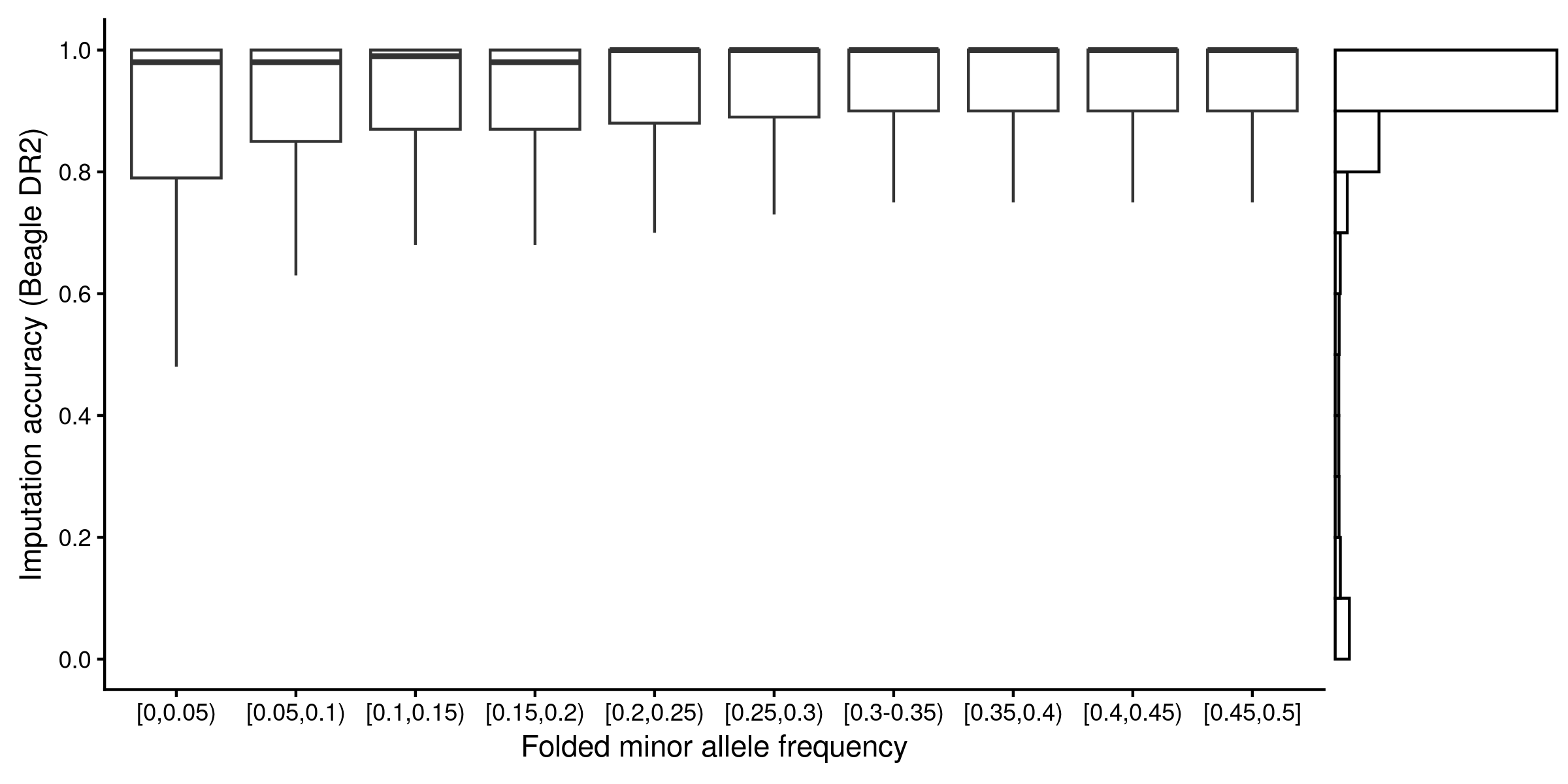


**Figure S7. Distribution of imputation accuracy values, as measured by Beagle Dosage R-Squared (DR2), for bins of folded minor allele frequency (MAF).** SNPs generally have high DR2 values, but SNPs with low MAF exhibit lower median DR2 and increased variation. The histogram on the right represents the distribution of DR2 values across all SNPs. SNPs with DR2 values lower than 0.9 were excluded from all analyses.


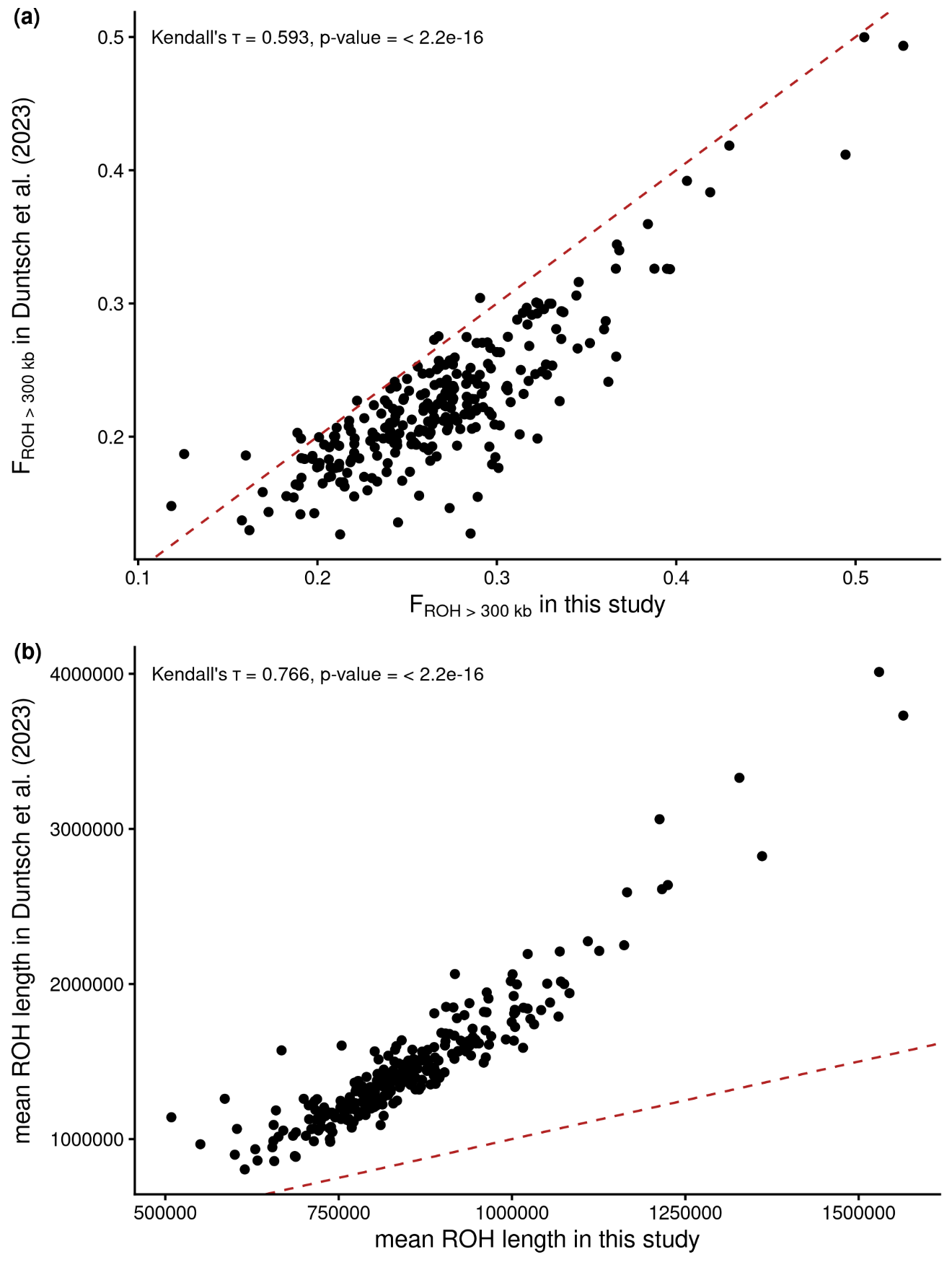


**Figure S8. Comparison of RZooRoH results between this study and Duntsch et al. (2023).** We compared (a) F_ROH > 300 kb_ and (b) mean ROH length for 268 individuals that were shared by both studies. In both plots, each point represents an individual, and the red dashed line represents a 1:1 correlation. Correlations between studies were tested for each value respectively using Kendall’s test (non-parametric). Both measurements were significantly correlated between the two studies, but Duntsch et al. (2023) found longer ROHs on average.

**
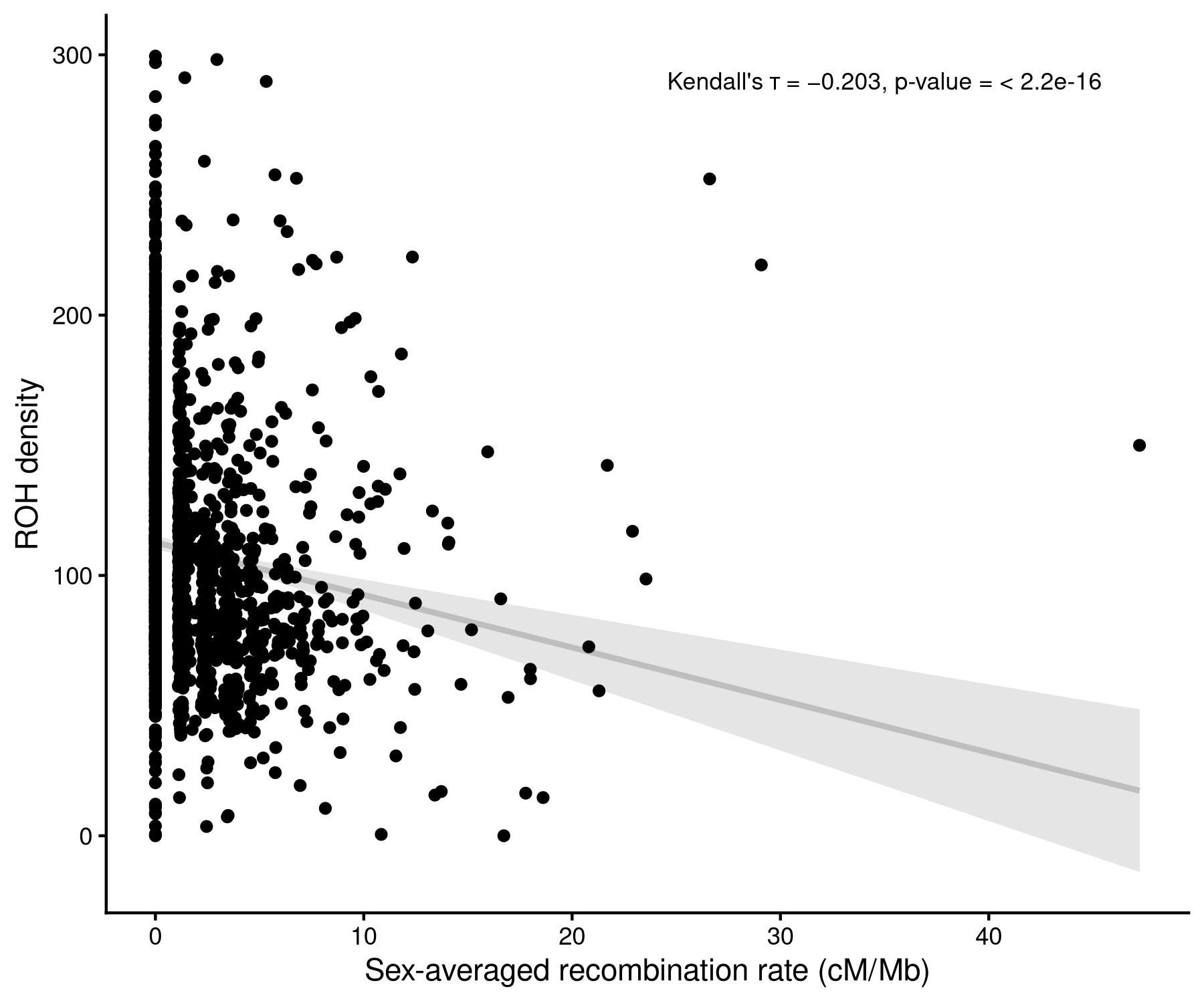
**

**Figure S9. ROH density vs sex-averaged recombination rate across the hihi genome.** Points on the figure represent non-overlapping 500 kb intervals. ROH density refers to the number of individuals in ROH in each interval. Kendall’s test (non-parametric) revealed significant, negative correlations between ROH density and sex-averaged recombination rate. The solid grey line represents the linear regression, and the shaded area represents the 95% confidence interval.


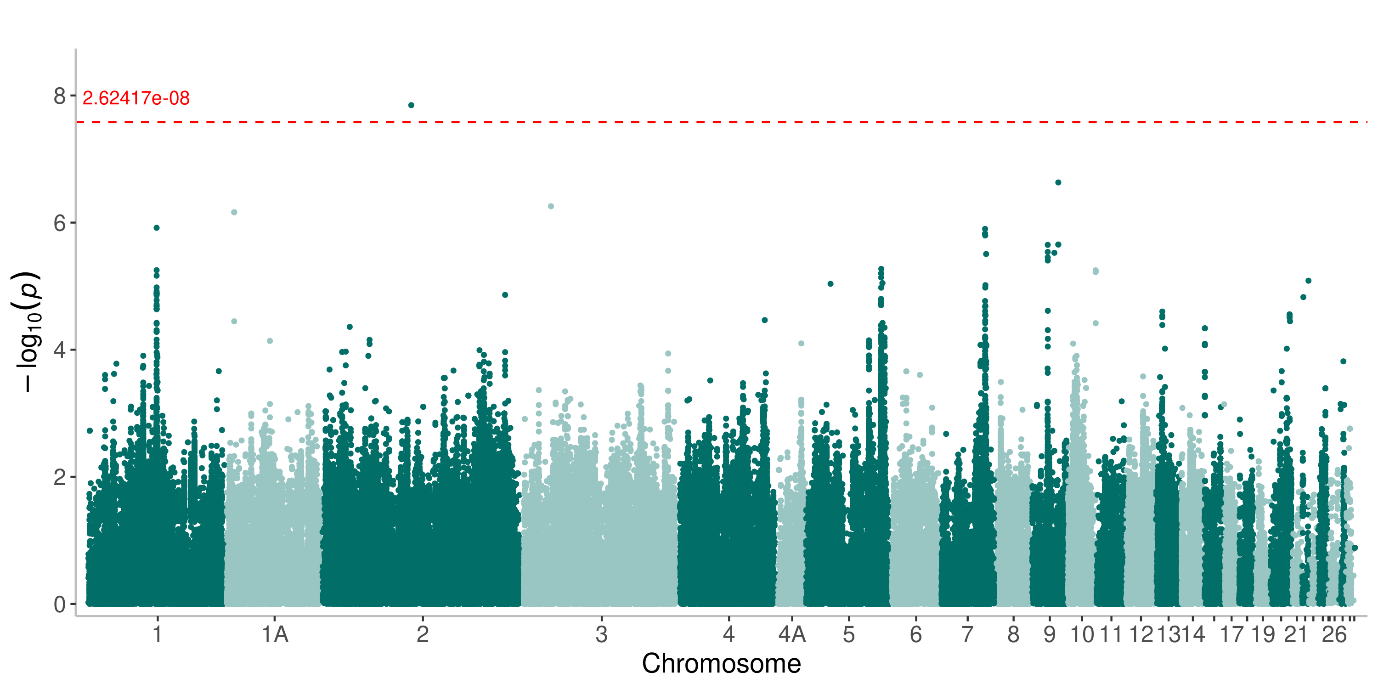


**Figure S10. Genome-wide association study (GWAS) of SNP effects on the lifetime reproductive success of 347 hihi / stitchbird individuals from Tiritiri Matangi using the additive model.** Lifetime reproductive success is the total number of offspring an individual has over its lifetime and was only calculated for individuals who have completed their reproductive lifespan. The red dashed line represents the Bonferroni-corrected significance threshold (−log10(p) > 7.62). Axis ticks after chromosome 21 represent chromosomes 22, 23, 24, 25A, 25B, 26 (labelled in figure), 27, 28, 29.

**
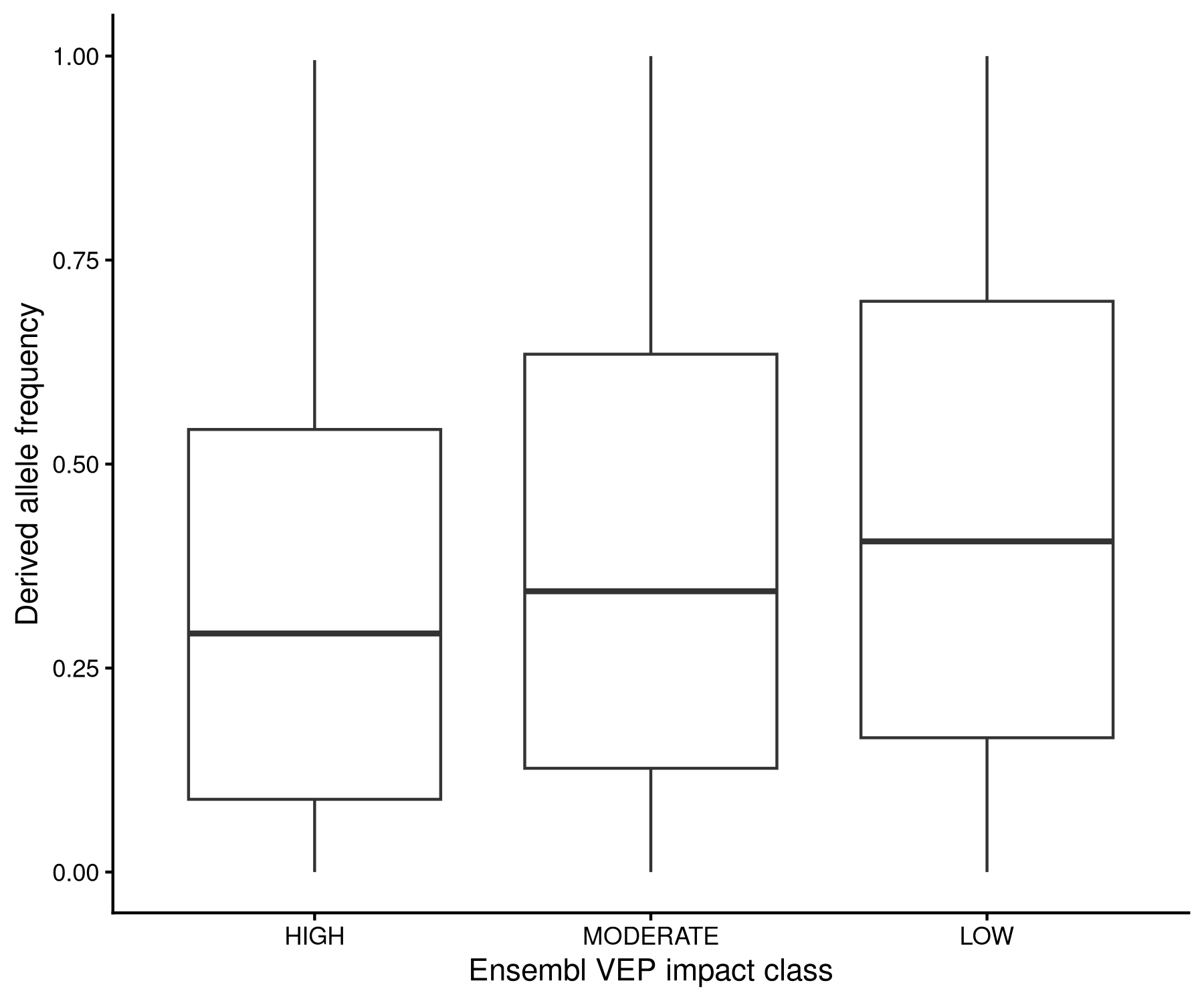
**

**Figure S11. Box plots of derived allele frequency of SNPs in respective Variant Effect Predictor (VEP) impact classes.** High-impact SNPs have the lowest median derived allele frequency, followed by moderate- then low-impact SNPs.


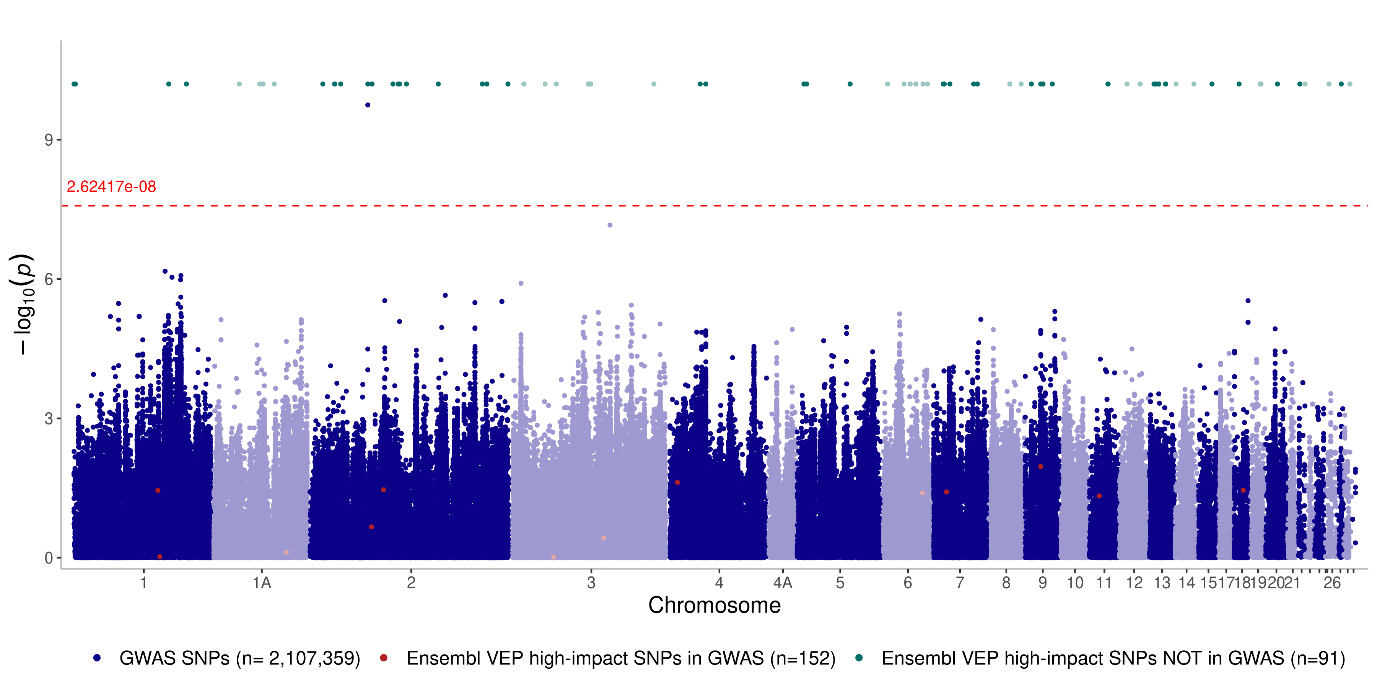


**Figure S12. Dominant GWAS with high-impact SNPs.** SNPs in blue represent the results of each SNP analysed in the TASSEL GWAS, while SNPs in red represent high-impact SNPs, predicted from Ensembl VEP, that were analysed in the GWAS. SNPs in green (at the top of the plot) were not included in the GWAS due to the applied MAF filter of 0.05 i.e., log likelihoods of green points were not calculated but are in a line at the top of the plot for visualisation purposes. We note a high-impact SNP (predicted as a stop gain/loss variant in the gene ATP2C1) close to the significant SNP on chromosome 2 (10,043 bp away). It was included in the GWAS analysis but subsequently removed from visualisation because it had only one individual with the rare homozygote genotype AA. Genotype effects from the dominance model were very similar for all genotype classes AA (1 individual), AC (251 individuals) and CC (95 individuals), and the p-value on the model was 0.701. Therefore, there was no evidence that this predicted high-impact SNP is causative.


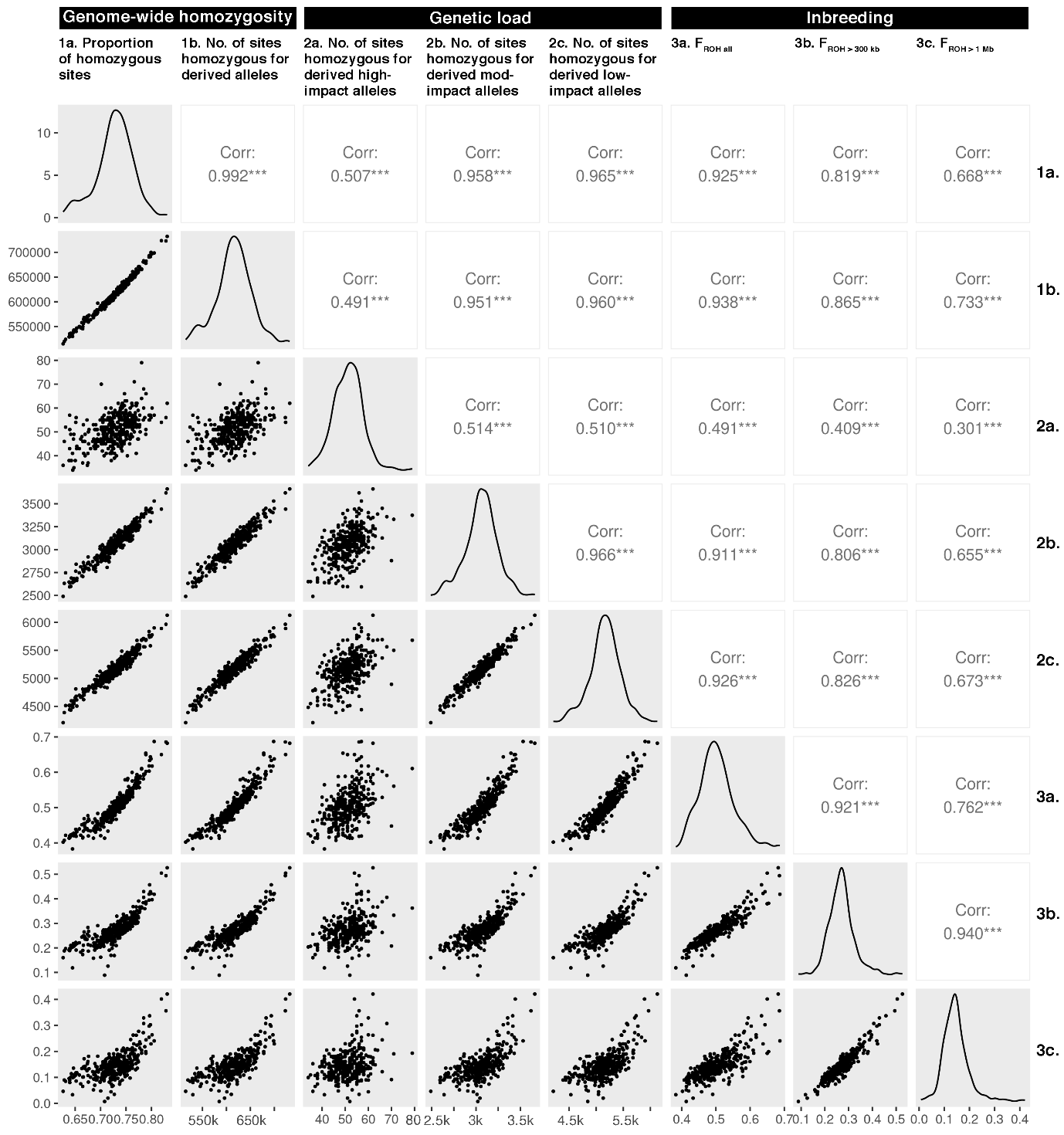


**Figure S13. Correlation between different measures of homozygosity, genetic load, and inbreeding.** Correlation results between two genome-wide homozygosity measures (proportion of homozygous SNPS, derived homozygosity count), 3 genetic load measures (derived homozygous counts of high-, moderate- (“mod-“), and low-impact SNPs) and 3 F_ROH_ measures (F_ROH all_, F_ROH > 300 kb_, F_ROH > 1 Mb_), as labelled at the top of the figure. Panels above the diagonal show correlation values; all correlations are highly significant p < 0.001, as indicated by ***. Density plots are on the diagonal, while plots below the diagonal show scatterplots. Labels on the right edge of the plot correspond to labels at the top of the figures.


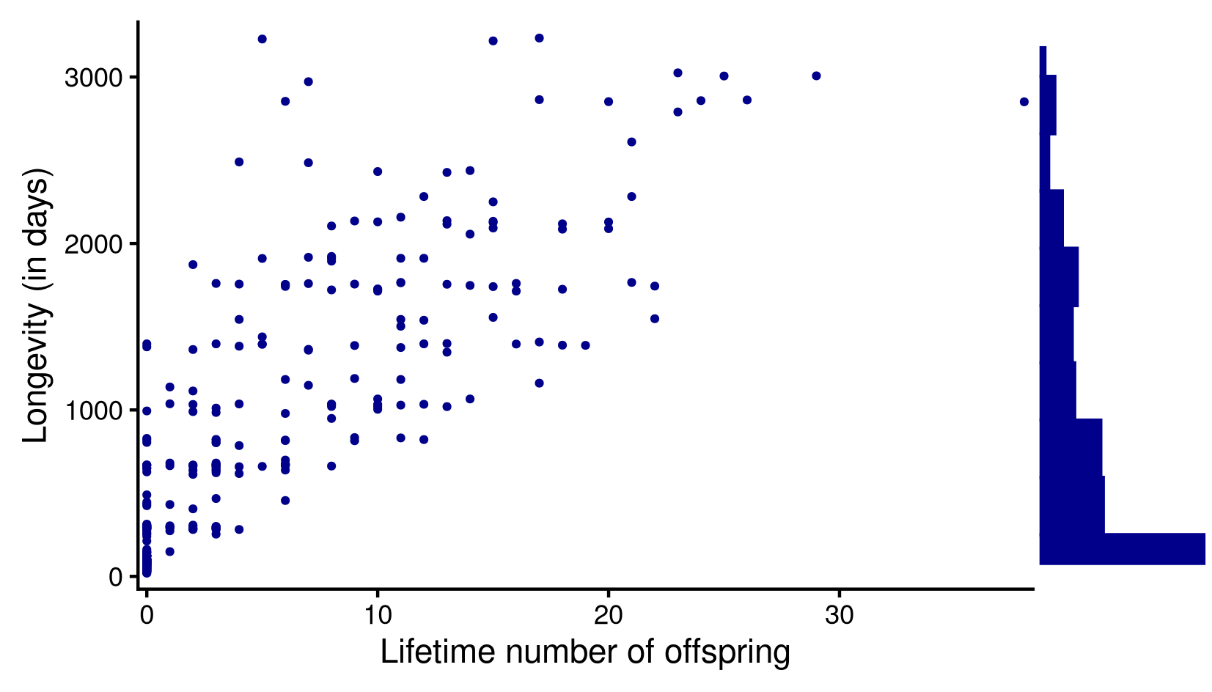


**Figure S14. Scatterplot of lifetime number of offspring against longevity (in days) of 347 hihi / stitchbird individuals from Tiritiri Matangi.** The total number of offspring per individual was only calculated for individuals who had completed their reproductive lifespan. The histogram along the vertical axis shows the distribution of longevity in the population.

**Table S1. Details of VEP input format for different SNP categories.** We divided our SNPs into four categories based on the inferred ancestral allele. We present here the proportion of SNPs in each category, along with the format used for input into VEP (AA = ancestral allele, REF = original reference allele, ALT = original alternate alleles).

| **Category** | **Description of category (proportion of SNPs)** | **Outcome** | **VEP input format** |
| --- | --- | --- | --- |
| 1 | AA match REF (54.7%) | retain ref/alt for input | REF/ALT |
| 2 | AA match ALT (35.7%) | swap the ref/alt bases for input | ALT/REF |
| 3 | no AA reconstructed (5.7%) | retain ref/alt for input | REF/ALT |
| 4 | novel AA (does not match REF or ALT; 3.9%) | split each position into two entries with the original ref/alt alleles as the alt alleles of each respective entry | AA/REF AA/ALT |

**Table S2.** **TASSEL results for the two most significant SNPs on chromosome 2 at 41,987,466 bp and on chromosome 3 at 70,449,698 bp.** The ‘Effect’ and ‘Obs’ columns represent the estimated effects of each genotype and the number of observations from the dataset. The remaining columns show the model statistics for the dominance model for each SNP.

| **Allele** | **Effect** | **Obs** | **dom_effect** | **dom_F** | **dom_p** | **errordf** | **MarkerR2** | **Genetic Var** | **Residual Var** | **-2LnLike-lihood** |
| --- | --- | --- | --- | --- | --- | --- | --- | --- | --- | --- |
| **Chr 2, 41,987,466 bp** | | | | | | | | | | |
| AA | -8.607 | 306 | 8.478 | 43.441 | 1.78E-10 | 324 | 0.122 | 2.63E-04 | 26.326 | 1974.029 |
| AG | 0.000 | 31 |  |  |  |  |  |  |  |  |
| GG | -8.348 | 10 |  |  |  |  |  |  |  |  |
| **Chr 3, 70,449,698 bp** | | | | | | | | | | |
| AA | -7.784 | 302 | 7.220 | 30.497 | 6.88E-08 | 324 | 0.082 | 2.81E-04 | 28.093 | 1995.015 |
| AC | 0.000 | 23 |  |  |  |  |  |  |  |  |
| CC | -6.657 | 22 |  |  |  |  |  |  |  |  |

**Table S3. MCMCglmm results using genotype at 41,987,466 bp on chromosome 2 as fixed effect for lifetime reproductive success (LRS).** The model was run using a zero-inflated Poisson error distribution with SNP genotype and sex as fixed factors. Maternal ID (dam_ID), cohort, and genetic relatedness (animal) were included as random effects. The model and resulting deviance information criterion (DIC) are provided in the first row. ‘traitLRS’ indicates the count component (non-zero-inflated) of the respective term, while ‘traitzi_LRS’ indicates the zero-inflated component. The first two terms represent the model intercept.

| LRS ~ trait - 1 + trait:genotype_2_41987466 + trait:sex,  random = ~ idh(trait):cohort + idh(trait):dam_ID + idh(trait):animal (DIC = 1361.294) | | | | | | |
| --- | --- | --- | --- | --- | --- | --- |
|  | **Post.mean** | **l-95% CI** | **u-95% CI** | **Eff.samp** | **pMCMC** | **effect** |
| traitLRS | 2.5918 | -43.3367 | 46.2288 | 46931.29 | 0.7542 | fixed |
| traitzi_LRS | -19.7904 | -184.778 | 135.3204 | 3582.157 | 0.6227 | fixed |
| traitLRS:genotype_2_41987466hom1 | -0.8428 | -1.1834 | -0.5108 | 4928.304 | <2e-05*** | fixed |
| traitzi_LRS:genotype_2_41987466hom1 | 19.0088 | 1.2665 | 56.7249 | 7.427 | <2e-05*** | fixed |
| traitLRS:genotype_2_41987466hom2 | -0.4434 | -1.2975 | 0.4208 | 18673.62 | 0.3096 | fixed |
| traitzi_LRS:genotype_2_41987466hom2 | 19.7697 | 0.9509 | 57.5544 | 6.81 | 0.0004*** | fixed |
| traitLRS:sex | -0.1216 | -0.37 | 0.1356 | 8727.595 | 0.3434 | fixed |
| traitzi_LRS:sex | -0.3022 | -1.026 | 0.4087 | 9324.461 | 0.4056 | fixed |
| traitLRS.cohort | 0.1138 | 2.20E-08 | 0.2749 | 17205 | NA | random |
| traitzi_LRS.cohort | 3.4549 | 0.2639 | 8.4761 | 2520 | NA | random |
| traitLRS.dam_ID | 0.1421 | 1.53E-08 | 0.307 | 8354 | NA | random |
| traitzi_LRS.dam_ID | 0.6815 | 2.44E-10 | 2.166 | 1418 | NA | random |
| traitLRS.animal | 0.02457 | 8.25E-11 | 0.08801 | 556.5 | NA | random |
| traitzi_LRS.animal | 0.3122 | 3.67E-11 | 1.23305 | 323.5 | NA | random |
| traitLRS.units | 0.3139 | 0.1657 | 0.4805 | 5554 | NA | residual |
| traitzi_LRS.units | 1 | 1 | 1 | 0 | NA | residual |

**Table S4. MCMCglmm results using genotype at 70,449,698 bp on chromosome 3 as fixed effect for lifetime reproductive success (LRS).** The model was run using a zero-inflated Poisson error distribution with SNP genotype and sex as fixed factors. Maternal ID (dam_ID), cohort, and genetic relatedness (animal) were included as random effects. The model and resulting deviance information criterion (DIC) are provided in the first row. ‘traitLRS’ indicates the count component (non-zero-inflated) of the respective term, while ‘traitzi_LRS’ indicates the zero-inflated component. The first two terms represent the model intercept.

| LRS ~ trait - 1 + trait:genotype_3_70449698 + trait:sex,  random = ~ idh(trait):cohort + idh(trait):dam_ID + idh(trait):animal (DIC = 1387.741) | | | | | | |
| --- | --- | --- | --- | --- | --- | --- |
|  | **Post.mean** | **l-95% CI** | **u-95% CI** | **Eff.samp** | **pMCMC** | **effect** |
| traitLRS | 2.819 | -37.46 | 45.71 | 51135 | 0.72932 | fixed |
| traitzi_LRS | -1.909 | -198.6 | 176 | 48863 | 0.93108 | fixed |
| traitLRS:genotype_3_70449698hom1 | -0.939 | -1.306 | -0.5624 | 36311 | <0.00002*** | fixed |
| traitzi_LRS:genotype_3_70449698hom1 | 1.63 | 0.006501 | 3.297 | 3044 | 0.03244* | fixed |
| traitLRS:genotype_3_70449698hom2 | -0.7938 | -1.378 | -0.2141 | 32836 | 0.00692** | fixed |
| traitzi_LRS:genotype__3_70449698hom2 | 1.236 | -0.8395 | 3.242 | 7601 | 0.21816 | fixed |
| traitLRS:sex | -0.1188 | -0.3692 | 0.1302 | 26342 | 0.3418 | fixed |
| traitzi_LRS:sex | -0.2552 | -0.9611 | 0.4479 | 12187 | 0.46316 | fixed |
| traitLRS.cohort | 0.1344 | 8.372E-09 | 0.3083 | 22127 | NA | random |
| traitzi_LRS.cohort | 4.4494 | 0.4532 | 11.0386 | 1023 | NA | random |
| traitLRS.dam_ID | 0.1114 | 6.75E-10 | 0.2565 | 11122 | NA | random |
| traitzi_LRS.dam_ID | 0.589 | 7.094E-11 | 1.9233 | 1051 | NA | random |
| traitLRS.animal | 0.02138 | 4.718E-11 | 0.07954 | 693.9 | NA | random |
| traitzi_LRS.animal | 0.49266 | 8.739E-11 | 2.08994 | 122.5 | NA | random |
| traitLRS.units | 0.3193 | 0.175 | 0.4802 | 14014 | NA | residual |
| traitzi_LRS.units | 1 | 1 | 1 | 0 | NA | residual |
|  |  |  |  |  |  |  |

**Table S5. Gene ontology (GO) terms overrepresented in 298 genes containing 1,460 strongly associated SNPs (p < 0.0001) from our GWAS with the dominant model.**

| **GO term** | **Ontology** | **Description** | **Fold enrichment** | **p-value** | **False discovery rate** |
| --- | --- | --- | --- | --- | --- |
| GO:1902731 | biological | negative regulation of chondrocyte proliferation | > 100 | 2.39E-07 | 2.90E-03 |
| GO:0005024 | molecular | transforming growth factor beta receptor activity | 68.15 | 8.23E-06 | 3.34E-02 |
| GO:0019199 | molecular | transmembrane receptor protein kinase activity | 11.93 | 1.06E-05 | 2.15E-02 |

**Table S6. GO terms overrepresented in 97 genes containing 258 SNPs predicted by Ensembl VEP to be high-impact.**

| **GO term** | **Ontology** | **Description** | **Fold enrichment** | **p-value** | **False discovery rate** |
| --- | --- | --- | --- | --- | --- |
| GO:0005515 | molecular | protein binding | 0.44 | 2.81E-18 | 1.33E-14 |

**Table S7. MCMCglmm results using genome-wide proportion of homozygous SNPs (“hom_prop”) as fixed effect for lifetime reproductive success (LRS).** All models (Tables S7–14) were run using a zero-inflated Poisson error distribution with a metric of homozygosity/genetic load/inbreeding, respectively, as well as sex as fixed effects. Maternal ID (dam_ID) and cohort were included as random effects. The model and resulting deviance information criterion (DIC) are provided in the first row. ‘traitLRS’ indicates the count component (non-zero-inflated) of the respective term, while ‘traitzi_LRS’ indicates the zero-inflated component. The first two terms represent the model intercept.

| LRS ~ trait - 1 + trait:hom_prop + trait:sex,  random = ~ idh(trait):cohort + idh(trait):dam_ID (DIC = 1382.566) | | | | | | |
| --- | --- | --- | --- | --- | --- | --- |
|  | **Post.mean** | **l-95% CI** | **u-95% CI** | **Eff.samp** | **pMCMC** | **effect** |
| traitLRS | 8.4316 | 6.3766 | 10.535 | 31695 | <2e-05*** | fixed |
| traitzi_LRS | -7.9631 | -15.1354 | -1.2176 | 10747 | 0.0239* | fixed |
| traitLRS:hom_prop | -8.9784 | -11.8528 | -6.0673 | 29949 | <2e-05*** | fixed |
| traitzi_LRS:hom_prop | 10.1651 | 0.5841 | 19.624 | 11493 | 0.0346* | fixed |
| traitLRS:sex | -0.1018 | -0.3359 | 0.1281 | 30476 | 0.3857 | fixed |
| traitzi_LRS:sex | -0.2575 | -0.8898 | 0.3924 | 13118 | 0.4277 | fixed |
| traitLRS.cohort | 0.0857 | 1.06E-08 | 0.206 | 22575 | NA | random |
| traitzi_LRS.cohort | 3.7269 | 3.98E-01 | 8.983 | 2124 | NA | random |
| traitLRS.dam_ID | 0.1005 | 4.91E-10 | 0.2309 | 11720 | NA | random |
| traitzi_LRS.dam_ID | 0.4214 | 5.76E-09 | 1.3997 | 3045 | NA | random |
| traitLRS.units | 0.302 | 0.1695 | 0.4475 | 15806 | NA | residual |
| traitzi_LRS.units | 1 | 1 | 1 | 0 | NA | residual |

**Table S8. MCMCglmm results using genome-wide count of homozygous derived alleles (“hom_derived_count”) as fixed effect for lifetime reproductive success (LRS).** All models (Tables S7–14) were run using a zero-inflated Poisson error distribution with a metric of homozygosity/genetic load/inbreeding, respectively, as well as sex as fixed effects. Maternal ID (dam_ID) and cohort were included as random effects. The model and resulting deviance information criterion (DIC) are provided in the first row. ‘traitLRS’ indicates the count component (non-zero-inflated) of the respective term, while ‘traitzi_LRS’ indicates the zero-inflated component. The first two terms represent the model intercept.

| LRS ~ trait - 1 + trait:hom_derived_count + trait:sex,  random = ~ idh(trait):cohort + idh(trait):dam_ID (DIC = 1383.637) | | | | | | |
| --- | --- | --- | --- | --- | --- | --- |
|  | **Post.mean** | **l-95% CI** | **u-95% CI** | **Eff.samp** | **pMCMC** | **effect** |
| traitLRS | 7.31E+00 | 5.50E+00 | 9.10E+00 | 28857 | <2e-05*** | fixed |
| traitzi_LRS | -6.13E+00 | -1.20E+01 | -4.63E-01 | 11619 | 0.0336* | fixed |
| traitLRS:hom_derived_count | -8.80E-06 | -1.17E-05 | -5.81E-06 | 27596 | <2e-05*** | fixed |
| traitzi_LRS:hom_derived_count | 9.03E-06 | -1.17E-07 | 1.83E-05 | 11813 | 0.0512. | fixed |
| traitLRS:sex | -1.01E-01 | -3.39E-01 | 1.32E-01 | 28031 | 0.3964 | fixed |
| traitzi_LRS:sex | -2.52E-01 | -8.97E-01 | 3.79E-01 | 13630 | 0.4411 | fixed |
| traitLRS.cohort | 0.08975 | 1.15E-11 | 0.2142 | 24073 | NA | random |
| traitzi_LRS.cohort | 3.75952 | 3.90E-01 | 8.8836 | 2056 | NA | random |
| traitLRS.dam_ID | 0.09777 | 7.19E-11 | 0.2315 | 10406 | NA | random |
| traitzi_LRS.dam_ID | 0.41371 | 2.38E-11 | 1.3768 | 3263 | NA | random |
| traitLRS.units | 0.3118 | 0.1756 | 0.4587 | 16357 | NA | residual |
| traitzi_LRS.units | 1 | 1 | 1 | 0 | NA | residual |

**Table S9. MCMCglmm results using with count of homozygous derived high-impact SNPs (“hom_derived_count_high”) as fixed effect for lifetime reproductive success (LRS).** All models (Tables S7–14) were run using a zero-inflated Poisson error distribution with a metric of homozygosity/genetic load/inbreeding, respectively, as well as sex as fixed effects. Maternal ID (dam_ID) and cohort were included as random effects. The model and resulting deviance information criterion (DIC) are provided in the first row. ‘traitLRS’ indicates the count component (non-zero-inflated) of the respective term, while ‘traitzi_LRS’ indicates the zero-inflated component. The first two terms represent the model intercept.

| LRS ~ trait - 1 + trait:hom_derived_count_high + trait:sex,  random = ~ idh(trait):cohort + idh(trait):dam_ID (DIC = 1390.112) | | | | | | |
| --- | --- | --- | --- | --- | --- | --- |
|  | **Post.mean** | **l-95% CI** | **u-95% CI** | **Eff.samp** | **pMCMC** | **Effect** |
| traitLRS | 3.86E+00 | 2.92E+00 | 4.79E+00 | 32360 | <2e-05*** | fixed |
| traitzi_LRS | 4.56E-01 | -2.27E+00 | 3.25E+00 | 14255 | 0.743 | fixed |
| traitLRS:hom_derived_count_high | -3.79E-02 | -5.65E-02 | -1.99E-02 | 30980 | <2e-05*** | fixed |
| traitzi_LRS:hom_derived_count_high | -2.08E-02 | -7.26E-02 | 2.97E-02 | 13367 | 0.419 | fixed |
| traitLRS:sex | -2.77E-02 | -2.80E-01 | 2.21E-01 | 30647 | 0.833 | fixed |
| traitzi_LRS:sex | -1.61E-01 | -7.85E-01 | 4.83E-01 | 14582 | 0.619 | fixed |
| traitLRS.cohort | 0.1093 | 6.70E-08 | 0.2597 | 23742 | NA | random |
| traitzi_LRS.cohort | 3.7407 | 5.48E-01 | 8.8494 | 2857 | NA | random |
| traitLRS.dam_ID | 0.08887 | 7.54E-13 | 0.2296 | 11520 | NA | random |
| traitzi_LRS.dam_ID | 0.39764 | 6.15E-14 | 1.3551 | 2882 | NA | random |
| traitLRS.units | 0.3767 | 2.24E-01 | 0.5446 | 17408 | NA | residual |
| traitzi_LRS.units | 1 | 1.00E+00 | 1 | 0 | NA | residual |

**Table S10. MCMCglmm results using count of homozygous derived moderate-impact SNPs (“hom_derived_count_mod”) as fixed effect for lifetime reproductive success (LRS).** All models (Tables S7–14) were run using a zero-inflated Poisson error distribution with a metric of homozygosity/genetic load/inbreeding, respectively, as well as sex as fixed effects. Maternal ID (dam_ID) and cohort were included as random effects. The model and resulting deviance information criterion (DIC) are provided in the first row. ‘traitLRS’ indicates the count component (non-zero-inflated) of the respective term, while ‘traitzi_LRS’ indicates the zero-inflated component. The first two terms represent the model intercept.

| LRS ~ trait - 1 + trait:hom_derived_count_mod + trait:sex,  random = ~ idh(trait):cohort + idh(trait):dam_ID (DIC = 1385.478) | | | | | | |
| --- | --- | --- | --- | --- | --- | --- |
|  | **Post.mean** | **l-95% CI** | **u-95% CI** | **Eff.samp** | **pMCMC** | **effect** |
| traitLRS | 7.10E+00 | 5.26E+00 | 8.91E+00 | 29606 | <2e-05*** | fixed |
| traitzi_LRS | -4.76E+00 | -1.05E+01 | 7.77E-01 | 12210 | 0.0939. | fixed |
| traitLRS:hom_derived_count_mod | -1.70E-03 | -2.30E-03 | -1.09E-03 | 28354 | <2e-05*** | fixed |
| traitzi_LRS:hom_derived_count_mod | 1.37E-03 | -4.73E-04 | 3.17E-03 | 12231 | 0.1412 | fixed |
| traitLRS:sex | -7.06E-02 | -3.09E-01 | 1.64E-01 | 29722 | 0.5592 | fixed |
| traitzi_LRS:sex | -2.48E-01 | -8.80E-01 | 3.94E-01 | 13859 | 0.4445 | fixed |
| traitLRS.cohort | 0.08991 | 2.36E-09 | 0.2186 | 22483 | NA | random |
| traitzi_LRS.cohort | 3.74863 | 4.56E-01 | 8.6605 | 2410 | NA | random |
| traitLRS.dam_ID | 0.1092 | 1.73E-11 | 0.2514 | 11044 | NA | random |
| traitzi_LRS.dam_ID | 0.3824 | 1.38E-09 | 1.2678 | 3446 | NA | random |
| traitLRS.units | 0.3154 | 1.77E-01 | 0.4677 | 16261 | NA | residual |
| traitzi_LRS.units | 1 | 1.00E+00 | 1 | 0 | NA | residual |

**Table S11. MCMCglmm results using count of homozygous derived low-impact SNPs (“hom_derived_count_low”) as fixed effect for lifetime reproductive success (LRS).** All models (Tables S7–14) were run using a zero-inflated Poisson error distribution with a metric of homozygosity/genetic load/inbreeding, respectively, as well as sex as fixed effects. Maternal ID (dam_ID) and cohort were included as random effects. The model and resulting deviance information criterion (DIC) are provided in the first row. ‘traitLRS’ indicates the count component (non-zero-inflated) of the respective term, while ‘traitzi_LRS’ indicates the zero-inflated component. The first two terms represent the model intercept.

| LRS ~ trait - 1 + trait:hom_derived_count_low + trait:sex,  random = ~ idh(trait):cohort + idh(trait):dam_ID (DIC = 1383.704) | | | | | | |
| --- | --- | --- | --- | --- | --- | --- |
|  | **Post.mean** | **l-95% CI** | **u-95% CI** | **Eff.samp** | **pMCMC** | **effect** |
| traitLRS | 7.48E+00 | 5.55E+00 | 9.43E+00 | 30475 | <2e-05*** | fixed |
| traitzi_LRS | -6.53E+00 | -1.27E+01 | -3.47E-01 | 12225 | 0.0354* | fixed |
| traitLRS:hom_derived_count_low | -1.08E-03 | -1.46E-03 | -6.98E-04 | 29208 | <2e-05*** | fixed |
| traitzi_LRS:hom_derived_count_low | 1.15E-03 | -4.38E-05 | 2.32E-03 | 12258 | 0.0541. | fixed |
| traitLRS:sex | -9.57E-02 | -3.32E-01 | 1.44E-01 | 30393 | 0.4262 | fixed |
| traitzi_LRS:sex | -2.66E-01 | -9.28E-01 | 3.51E-01 | 14081 | 0.4151 | fixed |
| traitLRS.cohort | 9.25E-02 | 7.26E-09 | 2.19E-01 | 24366 | NA | random |
| traitzi_LRS.cohort | 3.85E+00 | 4.47E-01 | 9.02E+00 | 2080 | NA | random |
| traitLRS.dam_ID | 1.06E-01 | 9.01E-10 | 2.44E-01 | 10879 | NA | random |
| traitzi_LRS.dam_ID | 3.94E-01 | 2.02E-10 | 1.31E+00 | 3620 | NA | random |
| traitLRS.units | 3.15E-01 | 1.80E-01 | 4.69E-01 | 16716 | NA | residual |
| traitzi_LRS.units | 1 | 1 | 1 | 0 | NA | residual |

**Table S12. MCMCglmm results using F_ROH all_ (“froh_all”) as fixed effect for lifetime reproductive success (LRS).** All models (Tables S7–14) were run using a zero-inflated Poisson error distribution with a metric of homozygosity/genetic load/inbreeding, respectively, as well as sex as fixed effects. Maternal ID (dam_ID) and cohort were included as random effects. The model and resulting deviance information criterion (DIC) are provided in the first row. ‘traitLRS’ indicates the count component (non-zero-inflated) of the respective term, while ‘traitzi_LRS’ indicates the zero-inflated component. The first two terms represent the model intercept.

| LRS ~ trait - 1 + trait:froh_all + trait:sex,  random = ~ idh(trait):cohort + idh(trait):dam_ID (DIC = 1386.329) | | | | | | |
| --- | --- | --- | --- | --- | --- | --- |
|  | **Post.mean** | **l-95% CI** | **u-95% CI** | **Eff.samp** | **pMCMC** | **effect** |
| traitLRS | 4.61E+00 | 3.41E+00 | 5.81E+00 | 28024 | <2e-05*** | fixed |
| traitzi_LRS | -2.7678 | -6.1818 | 0.6313 | 12920 | 0.11 | fixed |
| traitLRS:froh_all | -5.3475 | -7.7086 | -2.904 | 26110 | 0.00008*** | fixed |
| traitzi_LRS:froh_all | 4.2786 | -2.278 | 10.755 | 13451 | 0.194 | fixed |
| traitLRS:sex | -0.1134 | -0.3539 | 0.1419 | 31015 | 0.365 | fixed |
| traitzi_LRS:sex | -0.2379 | -0.8781 | 0.4021 | 14105 | 0.467 | fixed |
| traitLRS.cohort | 0.1022 | 3.01E-08 | 0.2435 | 23594 | NA | random |
| traitzi_LRS.cohort | 3.9825 | 5.03E-01 | 9.4504 | 2556 | NA | random |
| traitLRS.dam_ID | 0.1011 | 3.32E-11 | 0.249 | 11126 | NA | random |
| traitzi_LRS.dam_ID | 0.3796 | 1.61E-10 | 1.309 | 3408 | NA | random |
| traitLRS.units | 0.3611 | 0.2033 | 0.5235 | 16413 | NA | residual |
| traitzi_LRS.units | 1 | 1 | 1 | 0 | NA | residual |

**Table S13. MCMCglmm results using F_ROH > 300 kb_ (“froh_300kb”) as fixed effect for lifetime reproductive success (LRS).** All models (Tables S7–14) were run using a zero-inflated Poisson error distribution with a metric of homozygosity/genetic load/inbreeding, respectively, as well as sex as fixed effects. Maternal ID (dam_ID) and cohort were included as random effects. The model and resulting deviance information criterion (DIC) are provided in the first row. ‘traitLRS’ indicates the count component (non-zero-inflated) of the respective term, while ‘traitzi_LRS’ indicates the zero-inflated component. The first two terms represent the model intercept.

| LRS ~ trait - 1 + trait:froh_300kb + trait:sex,  random = ~ idh(trait):cohort + idh(trait):dam_ID (DIC = 1388.943) | | | | | | |
| --- | --- | --- | --- | --- | --- | --- |
|  | **Post.mean** | **l-95% CI** | **u-95% CI** | **Eff.samp** | **pMCMC** | **effect** |
| traitLRS | 3.1191 | 2.44 | 3.7889 | 29696 | <0.00002*** | fixed |
| traitzi_LRS | -1.3091 | -3.2894 | 0.4731 | 13305 | 0.16548 | fixed |
| traitLRS:froh_300kb | -4.4033 | -6.8117 | -1.9987 | 26938 | 0.00032*** | fixed |
| traitzi_LRS:froh_300kb | 2.5572 | -3.1839 | 8.6123 | 14302 | 0.39016 | fixed |
| traitLRS:sex | -0.109 | -0.3648 | 0.1386 | 30649 | 0.39548 | fixed |
| traitzi_LRS:sex | -0.2288 | -0.8766 | 0.4005 | 13876 | 0.48852 | fixed |
| traitLRS.cohort | 0.1101 | 3.13E-11 | 0.2585 | 23762 | NA | random |
| traitzi_LRS.cohort | 3.9335 | 4.46E-01 | 9.2026 | 2630 | NA | random |
| traitLRS.dam_ID | 0.07944 | 1.55E-11 | 0.219 | 11571 | NA | random |
| traitzi_LRS.dam_ID | 0.41394 | 9.32E-10 | 1.368 | 3385 | NA | random |
| traitLRS.units | 0.399 | 0.2409 | 0.5731 | 17784 | NA | residual |
| traitzi_LRS.units | 1 | 1 | 1 | 0 | NA | residual |

**Table S14. MCMCglmm results using F_ROH > 1 Mb_ (“froh_1Mb”) as fixed effect for lifetime reproductive success (LRS).** All models (Tables S7–14) were run using a zero-inflated Poisson error distribution with a metric of homozygosity/genetic load/inbreeding, respectively, as well as sex as fixed effects. Maternal ID (dam_ID) and cohort were included as random effects. The model and resulting deviance information criterion (DIC) are provided in the first row. ‘traitLRS’ indicates the count component (non-zero-inflated) of the respective term, while ‘traitzi_LRS’ indicates the zero-inflated component. The first two terms represent the model intercept.

| LRS ~ trait - 1 + trait:froh_1Mb + trait:sex,  random = ~ idh(trait):cohort + idh(trait):dam_ID (DIC = 1390.015) | | | | | | |
| --- | --- | --- | --- | --- | --- | --- |
|  | **Post.mean** | **l-95% CI** | **u-95% CI** | **Eff.samp** | **pMCMC** | **effect** |
| traitLRS | 2.4282 | 1.9912 | 2.8645 | 31165 | <2e-05*** | fixed |
| traitzi_LRS | -0.9296 | -2.3309 | 0.4507 | 11024 | 0.1746 | fixed |
| traitLRS:froh_1Mb | -3.4136 | -6.0122 | -0.7869 | 28496 | 0.0108* | fixed |
| traitzi_LRS:froh_1Mb | 2.1904 | -4.0009 | 8.5967 | 13720 | 0.4884 | fixed |
| traitLRS:sex | -0.1044 | -0.3597 | 0.1493 | 31576 | 0.4209 | fixed |
| traitzi_LRS:sex | -0.225 | -0.8907 | 0.3873 | 14630 | 0.4834 | fixed |
| traitLRS.cohort | 0.1337 | 1.52E-08 | 0.3049 | 25317 | NA | random |
| traitzi_LRS.cohort | 3.9412 | 4.98E-01 | 9.3465 | 1960 | NA | random |
| traitLRS.dam_ID | 0.06847 | 1.77E-10 | 0.1994 | 13726 | NA | random |
| traitzi_LRS.dam_ID | 0.39097 | 1.28E-09 | 1.3396 | 3500 | NA | random |
| traitLRS.units | 0.428 | 0.2646 | 0.6066 | 19478 | NA | residual |
| traitzi_LRS.units | 1 | 1 | 1 | 0 | NA | residual |
